# Effects of type of substrate and dilution rate on fermentation in serial rumen mixed cultures

**DOI:** 10.3389/fmicb.2024.1356966

**Published:** 2024-02-09

**Authors:** Emilio M. Ungerfeld, Nathaly Cancino-Padilla, Nelson Vera-Aguilera, M. Carolina Scorcione, Marcelo Saldivia, Lorena Lagos-Pailla, Milena Vera, Cristián Cerda, Camila Muñoz, Natalie Urrutia, Emilio D. Martínez

**Affiliations:** ^1^Centro Regional de Investigación Carillanca, Instituto de Investigaciones Agropecuarias, Vilcún, Chile; ^2^Facultad de Agronomía, Universidad de Buenos Aires, Buenos Aires, Argentina; ^3^Instituto de Ciencia Animal, Facultad de Ciencias Veterinarias, Universidad Austral de Chile, Valdivia, Chile; ^4^Instituto de Ingeniería Agraria y Suelos, Facultad de Ciencias Agrarias y Alimentarias, Universidad Austral de Chile, Valdivia, Chile; ^5^Centro de Investigación de Suelos Volcánicos, Universidad Austral de Chile, Valdivia, Chile; ^6^Centro de Humedales Río Cruces, Valdivia, Chile; ^7^Departamento de Procesos Industriales, Universidad Católica de Temuco, Temuco, Chile; ^8^Centro Regional de Investigación Remehue, Instituto de Investigaciones Agropecuarias, Osorno, Chile

**Keywords:** rumen fermentation, dihydrogen, type of substrate, dilution rate, methane, methanogens, volatile fatty acids, lactate

## Abstract

Forages and concentrates have consistently distinct patterns of fermentation in the rumen, with forages producing more methane (CH_4_) per unit of digested organic matter (OM) and higher acetate to propionate ratio than concentrates. A mechanism based on the Monod function of microbial growth has been proposed to explain the distinct fermentation pattern of forages and concentrates, where greater dilution rates and lower pH associated with concentrate feeding increase dihydrogen (H_2_) concentration through increasing methanogens growth rate and decreasing methanogens theoretically maximal growth rate, respectively. Increased H_2_ concentration would in turn inhibit H_2_ production, decreasing methanogenesis, inhibit H_2_-producing pathways such as acetate production via pyruvate oxidative decarboxylation, and stimulate H_2_-incorporating pathways such as propionate production. We examined the hypothesis that equalizing dilution rates in serial rumen cultures would result in a similar fermentation profile of a high forage and a high concentrate substrate. Under a 2 × 3 factorial arrangement, a high forage and a high concentrate substrate were incubated at dilution rates of 0.14, 0.28, or 0.56 h^−1^ in eight transfers of serial rumen cultures. Each treatment was replicated thrice, and the experiment repeated in two different months. The high concentrate substrate accumulated considerably more H_2_ and formate and produced less CH_4_ than the high forage substrate. Methanogens were nearly washed-out with high concentrate and increased their initial numbers with high forage. The effect of dilution rate was minor in comparison to the effect of the type of substrate. Accumulation of H_2_ and formate with high concentrate inhibited acetate and probably H_2_ and formate production, and stimulated butyrate, rather than propionate, as an electron sink alternative to CH_4_. All three dilution rates are considered high and selected for rapidly growing bacteria. The archaeal community composition varied widely and inconsistently. Lactate accumulated with both substrates, likely favored by microbial growth kinetics rather than by H_2_ accumulation thermodynamically stimulating electron disposal from NADH into pyruvate reduction. In this study, the type of substrate had a major effect on rumen fermentation largely independent of dilution rate and pH.

## 1 Introduction

Ruminants play an important role in global agriculture due to their ability to convert feedstuffs unusable to humans into meat, milk, and other useful products. The complex microbial community inhabiting their rumen can digest fiber, synthesize amino acids from non-protein nitrogen, and synthesize water-soluble vitamins. Volatile fatty acids (VFA) resulting from feed digestion and fermentation are absorbed through the rumen wall and utilized by the host animal as a source of energy, carbon for diverse metabolites, and glucose, while microbial cells formed in the rumen are distally digested in the gastrointestinal tract to provide amino acids and other nutrients. Forages and concentrates have consistently distinct fermentation patterns in the rumen. Replacing forages by concentrates shifts rumen fermentation from acetate to propionate and produces less CH_4_ per unit of organic matter (OM) fermented (Janssen, 2010). This has environmental implications, as CH_4_ is a potent greenhouse gas second to carbon dioxide (CO_2_) in its contribution to global warming. Mitigating anthropogenic CH_4_ emissions is regarded as key for short-term amelioration of global warming due to the relatively short lifetime of CH_4_ in the atmosphere (Connors et al., 2021; Szopa et al., 2021). In addition, the release of CH_4_ produced in the rumen to the atmosphere is a loss of energy ranging between 2% and 12% of gross energy ingested by ruminants (Beauchemin et al., 2020). Also, the profile of volatile fatty acids absorbed from the rumen has consequences for animal metabolism. Acetate is oxidized in animal tissues to generate ATP and used as a carbon skeleton in the synthesis of long chain fatty acids, while propionate is the main glucogenic precursor in ruminants (van Houtert, 1993; Loncke et al., 2020). Understanding the mechanisms controlling rumen fermentation is important for both mitigating CH_4_ emissions and improving animal productivity.

Cellulose is the predominant carbohydrate polymer in forages and starch predominates in concentrates. The same as forages and concentrates, the digestion and fermentation of cellulose also results in more CH_4_ and a higher acetate to propionate ratio than starch (Czerkawski, 1969; Janssen, 2010; Ungerfeld et al., 2020). Cellodextrins, cellobiose, and β-glucose resulting from cellulose digestion in the rumen are taken up by both cellulolytic and by non-cellulolytic organisms that cross-feed on them (Russell, 1985). Inside cells, hydrolytic and phosphorolytic cleavage of cellodextrins and cellobiose yields β-glucose and glucose-1-phosphate (Lynd et al., 2002). Similarly, rumen bacteria digest starch to maltodextrins, maltose, and α-glucose (Cotta, 1988), which are also taken up by non-amylolytic species (Cotta, 1992). Protozoa engulf starch and take up maltose and glucose, metabolizing them to glucose and glucose-6-phosphate (Coleman and Laurie, 1976, 1977). The main route of glucose metabolism is glycolysis, with pyruvate and phosphoenolpyruvate being the central branching points at which the different pathways of VFA formation diverge (Russell and Wallace, 1997; Russell, 2002).

Therefore, the consistent differences in fermentation profile between cellulose and starch do not seem to be explained by differences in the monomers presented to catabolism. Janssen (2010) proposed a mechanistic explanation based on rumen passage rate and pH influence methanogens growth kinetics and in turn dihydrogen (H_2_) concentration, explaining the distinctive fermentation patterns of forages and concentrates. Typically, concentrates pass out of the rumen faster than do forages because they are digested and fermented faster. According to the Monod function of microbial growth, rapid growth of methanogens required to match the rapid rumen passage rates of concentrates will occur at elevated H_2_ concentration (Janssen, 2010), considering H_2_ as the main electron donor for rumen methanogenesis (Hungate, 1967). Elevated concentration of H_2_ would in turn thermodynamically favor a shift from H_2_-producing pathways such as acetate production, to H_2_-incorporating pathways such as propionate production. Through decreasing H_2_ production, elevated H_2_ concentration is proposed to decrease CH_4_ production. Apart from passage rate, the lower pH associated to the rapid fermentation of concentrates in the rumen in comparison to forages, is proposed as an additional mechanism explaining the distinct fermentation profiles of forages and concentrates through decreasing methanogens theoretically maximal rate of growth, also elevating H_2_ concentration (Janssen, 2010).

In view of the above rationale, we hypothesized that, if dilution rates are equalized in serially transferred rumen mixed cultures adequately buffered so that pH is not allowed to fall to levels negatively affecting methanogens growth, a high forage and a high concentrate substrate would respond similarly in accumulation of H_2_, production of CH_4_, and the acetate to propionate ratio. The high forage and the high concentrate substrate would be expected to equally increase H_2_ accumulation and decrease CH_4_ production and the acetate to propionate ratio as dilution rate increases. Our objective was to study the effects of the type of substrate, dilution rate, and their interaction, on the evolution of the fermentation profile and composition of prokaryotic communities in serially transferred rumen mixed cultures.

## 2 Materials and methods

All animal procedures were approved by Instituto de Investigaciones Agropecuarias’s Comité Institucional de Cuidado Animal (Approval number 02/2019 from 30 June 2019).

### 2.1 Treatments and incubations

**Figure 1:**
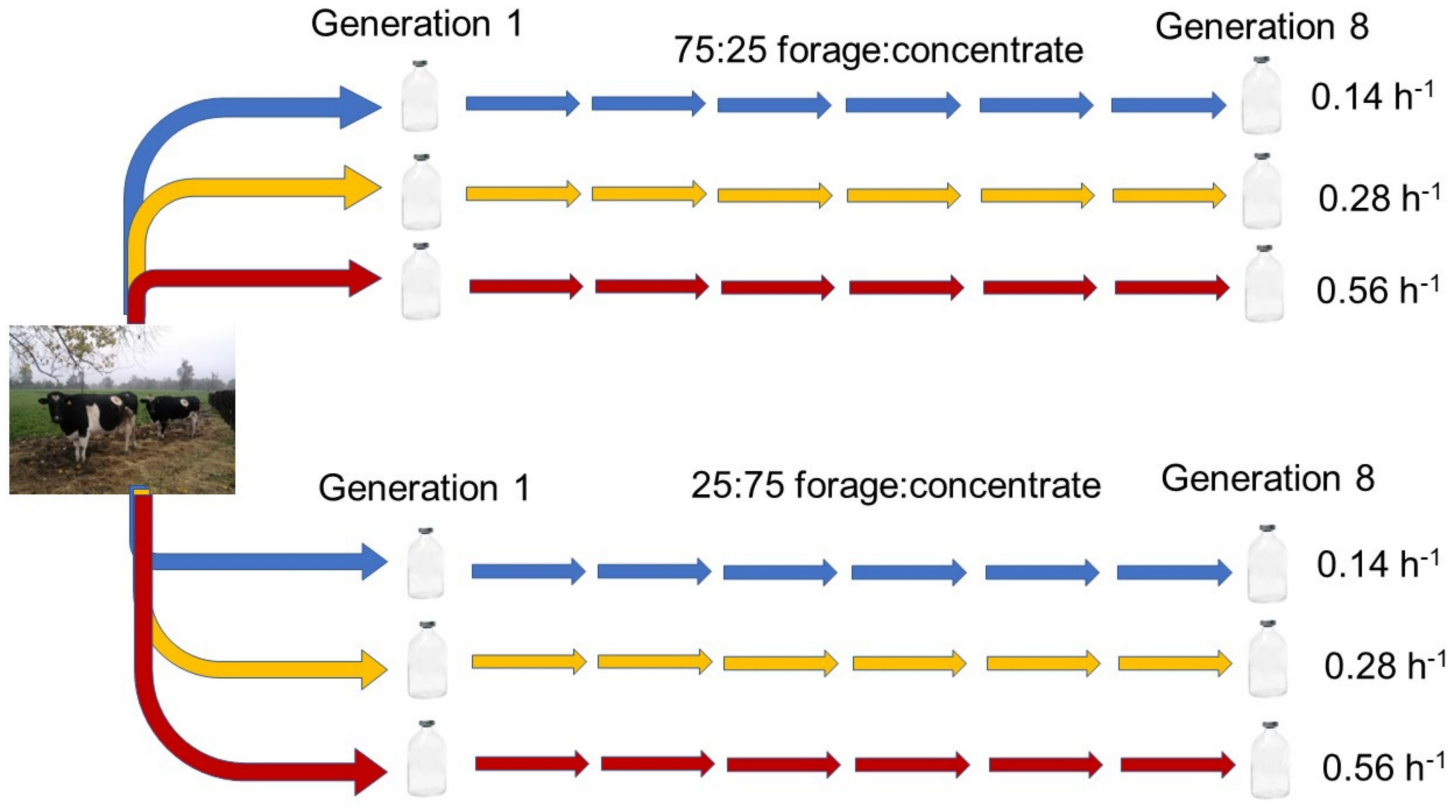
Scheme depicting rumen mixed culture incubations with a high forage or a high concentrate substrate, each transferred through eight serial 72 h-incubations at three average dilution rates of 0.14 (low), 0.28 (medium), or 0.56 (high) h^−1^.

The rumen serial culture experiment was conducted as a 2 × 3 factorial arrangement of treatments, with two isonitrogenous high forage (75:25 forage: concentrate, DM basis) or high concentrate (25:75 forage: concentrate, DM basis) substrates (Supplementary Table S1) incubated at three different average dilution rates (0.14, 0.28, and 0.56 h^−1^). Differing dilution rates were obtained through transferring different volumes of inoculum from donor to receiving incubation bottles in serial rumen cultures, with all cultures growing for the same 72-h time interval until inoculating the next bottle in the serial culture (Figure 1). In this regard, the term “dilution rate” used herein does not correspond to the classic definition of a constant dilution rate in a chemostat, but to average transfer rates across sequential incubation bottles in the serially transferred rumen culture. Because a secondary objective of this study was to identify for a forthcoming experiment the minimal volume of inoculum compatible with maintaining functional methanogenesis, relatively small inoculation volumes and hence fast dilution rates were used.

Rumen contents were sampled shortly after the morning from two ruminally cannulated non-lactating, non-pregnant, Holstein cows, fed about 9 kg/d DM ryegrass hay (DM, 88.4%, and on a DM basis CP, 6.3%, NDF, 68.0%, total ash, 6.0%), and strained through a 2-layer synthetic cloth (~0.5 mm mesh; Eurotelas, Osorno, Chile). Rumen fluid from both cows was pooled and immediately transported to the laboratory in an insulated flask, while solids from each cow were transported in separate insulated flasks.

All laboratory procedures involving rumen fluid and solids, rumen inoculum, and inoculated culture medium, took place under O_2_-free CO_2_. About 200 mL of rumen fluid were transferred to a 500-mL Erlenmeyer. Rumen solids from both cows were added in approximately equal volumes until reaching about 400 mL, and the resulting recomposed rumen contents were blended discontinuously for 1 min (3 s bursts followed by 2 s pauses) to detach microbes adhered to plant particles. Blended rumen contents were then strained through a 2-layer synthetic cloth to obtain the rumen inoculum.

The three different average dilution rates treatments were initiated in transfer 1 by inoculating 100-mL serum bottles containing 39, 38, or 36 mL of the incubation medium by Mould et al. (2005), as modified by Raju (2016) (Supplementary Table S2), with 1, 2, or 4 mL of rumen inoculum. Average dilution rate treatments initiated in transfer 1 were maintained throughout the serial incubation by inoculating the same volumes of microbial culture from an incubation bottle into fresh medium at the end of each 72-h batch incubation of the serial culture (Figure 1). Bottles contained 401 ± 0.60 (mean ± SD) mg of finely ground (1-mm screen) high forage or high concentrate substrate (Supplementary Table S1). Samples of the initial rumen inoculum and the incubation medium were taken and stored at −20°C for subsequent analysis of concentration of VFA and ammonium (NH_4_^+^). Samples of the initial rumen inoculum were also stored at −20°C for subsequent analysis of their bacterial and archaeal community abundance and composition.

Inoculated bottles were sealed under O_2_-free CO_2_ and incubated at 39°C and 60 rpm oscillation for 72 h. At the end of the incubations, gas pressure was measured with a pressure transducer (Sper Scientific 840,065, Scottsdale, AZ, United States), and 30-mL (ambient pressure) gas samples were taken with a syringe and entirely delivered into 5.9-mL previously evacuated exetainers (Labco Limited, Lampeter, Ceredigion, United Kingdom) to over pressurize them. Bottles were then vortexed for 2 min to detach microbes adhered to the undigested solid residue, and thereafter left still for 5 min to allow solid particles to sediment. Next, bottles were uncapped under O_2_-free CO_2_, and 1, 2, or 4 mL of fluid from each bottle that had received the corresponding volume of initial inoculum, was delivered as inoculum to serum bottles containing 39, 38, or 36 mL, respectively, of fresh growth medium and the same type of substrate. The newly inoculated bottles were capped under O_2_-free CO_2_ and incubated at 39°C and 60 rpm oscillation for 72 h. The pH (Orion Star A214, Thermo Scientific, Chelmsford, MA, United States) and reducing potential (E*h*; Oakton pH 700 meter, Vernon Hills, IL, United States, Ag/AgCl electrode in saturated KCl, Schott Instruments, BlueLine 31 Rx, Mainz, Germany) were immediately measured in the uncapped previous transfer bottles from which the microbial inoculum had been taken.

The serial inoculation process was repeated eight times from the initial inoculation of transfer 1 (first batch cultures) to transfer 8 (final batch cultures). In each transfer, after inoculation of the next incubation bottles, 1 mL of fluid was sampled and delivered into 2-mL microcentrifuge tubes containing 0.2 mL of 20% (*V*/*V*) *o*-phosphoric acid for subsequent analysis of VFA, formate, lactate, and succinate concentration. A second 1-mL aliquot was sampled and delivered into 2-mL microcentrifuge tubes containing 0.2 mL of 1% sulfuric acid for determination of ammonium (NH_4_^+^) concentration. All samples were stored at −20°C until analyzed.

The experiment was first conducted in December 2021 (incubation 1) and repeated in February 2022 (incubation 2). Whole bottle contents from transfer 8 in both incubation 1 and 2 and also from transfer 4 in incubation 2, were centrifuged at 10,956 × *g* and 4°C for 15 min in pre-weighted centrifuge tubes. The supernatant was discarded, and the pellet was frozen at −80°C, and subsequently lyophilized to obtain a residue composed by undigested substrate and microbial biomass. Lyophilized tubes were weighted, and the residue dry mass calculated by difference.

### 2.2 Analytical procedures

Gas samples were analyzed for concentration of CH_4_ and H_2_ in a Clarus 580 Perkin Elmer GC equipped with a 60/80 Carboxen 1,000 (Supelco, Bellefonte, PA, United States), as previously described (Ungerfeld et al., 2020). Concentration of CH_4_ and H_2_ was obtained through calibration against known standards. Concentration of CH_4_ and H_2_ was then adjusted for residual air remaining in the exetainers when delivering gas samples; residual air in the exetainers was determined through oxygen concentration measured in the GC divided by 0.2095 [i.e., the molar proportion of oxygen in the atmosphere (Allen, 1973)]. Volatile fatty acids samples were thawed, vortexed, centrifuged at 16,100 × g and 4°C for 10 min, and filtered through 0.45 μm pore filters into 2 mL GC vials, and analyzed for VFA concentration by GC (PerkinElmer Clarus 580) as done previously (Ungerfeld et al., 2020). Samples used for VFA analysis were subsequently analyzed for concentration of formate, lactate, and succinate by injecting 20 μL into an LC-20A Shimadzu HPLC (Kyoto, Japan) equipped with a Kromasil RP-18e (5 μm, 300 × 4.6 mm) column (Bohus, Sweden) and a Shimadzu SPD-M20A Photodiode Array Detector (Kyoto, Japan). The mobile phase was 0.1% (*V*/*V*) *o*-phosphoric acid at a ramp from 0.2 to 1 mL/min for a 20 min run. Concentration of NH_4_^+^ was analyzed by colorimetry according to Kaplan (1969).

### 2.3 Carbon-13 tracer study

In order to investigate the possibility that increased H_2_ accumulation might stimulate the incorporation of bicarbonate carbon into VFA via acetyl-CoA (Ragsdale and Pierce, 2008), two of the three replicates per combination of substrate and dilution rate in incubation 2, transfer 8, were enriched with ^13^C-labeled bicarbonate (Cambridge Isotope Laboratories, Inc., Andover, MS, United States), to achieve a 5% enrichment above natural ^13^C abundance in total bicarbonate (Raju, 2016). One bottle per treatment combination of substrate and dilution rate was left as a standard to determine ^13^C natural abundance and received an equimolar amount of non-enriched bicarbonate. At the end of the incubation, 1.1 mL aliquots of incubation fluid were delivered into microcentrifuge tubes and processed for GC-MS analysis following the method by Richardson et al. (1989). Samples were acidified with 0.25 mL of concentrated hydrochloric acid and centrifuged at 7,200 × *g* for 30 min. Following, 0.5 mL of supernatants were filtered through a 0.25 μm syringe filter into a new microcentrifuge tube, and 0.70 mL of ethyl ether added. Samples with ether were then vortexed for 1 min and thereafter centrifuged at 3,500 × *g* for 10 min to separate an aqueous and an ether layer containing the protonated VFA. Next, 0.20 mL of the ether upper layer were removed and delivered into a glass V-vial, and 0.30 mL of N-(tert.-butyldimethylsilyl)-N-methyl-trifluoroacetamide (MTBSTFA) derivatizing agent added. Vials were heated at 80°C for 20 min, and after cooling down, 0.10 mL of methanol were added.

The resulting V-vial contents were then transferred to GC vials, and 1 μL was injected into a Shimadzu QP2010 plus GC–MS (Kyoto, Japan) equipped with an InertCap 5 ms-Sil (30 m, 0.25 mm, 0.25 mm) capillary column (GL Sciences, Torrance, CA, United States). The initial oven temperature was 50°C for 4 min, it was then ramped to 105°C at 5°C/min, and then ramped to a final temperature of 250°C at 20°C/min at which was held for 5 min. Helium at 1.47 mL/min was used as carrier gas. The electron ionization mass spectrometer operated in FASST mode, with a scan mode operating at a mass/charge (*m*/*z*) range between 35 and 400. Ion source was operated at 250°C.

### 2.4 DNA extraction and bacterial and archaeal qPCR

Genomic DNA was extracted following the procedure by Yu and Forster (2005) from lyophilized samples of incubation 1, transfer 8, from incubation 2, transfers 4 and 8, and from the initial inocula of incubations 1 and 2 (total of 56 samples: 2 types of substrate × 3 dilution rates × 3 replicates × 3 combinations of incubation-transfer numbers +2 initial inocula). Approximately 100 mg of lyophilized residues were used for DNA extraction with repeated bead-beating (Yu and Forster, 2005). The DNA concentration and quality were checked through their A260 to A280 absorbance ratio (1.64 ± 0.24; mean ± SD) using a Maestrogen Spectrophotometer (Maestrogen, Hsinshu City, Taiwan). The gDNA extracts were stored at −20°C until qPCR and 16S rRNA gene sequencing analyses.

The abundance of total bacteria and archaea was estimated in 50 μL aliquots containing approximately 100 ng/μL gDNA through quantitative polymerase chain reaction (qPCR) of 16S RNA and *mcrA* genes, respectively, using a Quant Studio™ 3 Real-Time PCR System (ThermoFisher Scientific, Inc., Waltham, MA, United States) and a PowerUp™ SYBR™ Green Master Mix (Applied Biosystems TM, Foster City, CA, United States). Primers sets for 16S rRNA bacterial (Maeda et al., 2003) and *mcrA* archaeal (Denman et al., 2007) genes and PCR conditions are shown in Supplementary Table S3. Abundance of total bacteria and archaea expressed as gene copies per gram of lyophilized cultures were calculated using standards of each gene built with dsDNA gBlock® Gene Fragments (Integrated DNA Technologies, Inc. Iowa, United States) following (Whelan et al., 2003):


$$\begin{array}{l} \text{copies}\;\left(\frac{\text{gene}\;\text{copies}}{g\;\text{lyophilized}\;\text{rumen}\;\text{culture}}\right)=\\ \left[\text{concentration}\;\text{of}\;\text{the}\;\text{dsDNA}\;\text{gBlock}\;\text{gene}\;\text{fragment}\;\left(\frac{\text{ng}}{\mathrm{\mu l}}\right)\right]\\ \times \left[\text{molar}\;\text{mass}\;\left(\frac{\text{fmol}}{\text{ng}}\right)\right]\times \left[{\text{Avogadro}}^{\prime }s\;\text{number}\right]\\ \times \left[\frac{\text{lyophilized}\;\text{residue}\;\text{aliquot}\;\left(\text{mg}\right)}{100\;\text{mg}}\right] \end{array}$$

Gene copies of total bacteria and archaea were also calculated on a per bottle basis by adjusting by the mass of the lyophilized incubation residue.

### 2.5 Bacterial and archaeal 16S rRNA gene sequencing

All samples were diluted to 18 ng DNA/μL. Amplicons for generating libraries for sequencing were produced following the Illumina 16S Metagenomic Sequencing Library Preparation protocol (Illumina Inc, 2013). Negative non-template controls were included in amplification reactions. Primers used for amplification of V4 region of bacterial and archaeal 16S rRNA genes are shown in Supplementary Table S4. Amplification of DNA was performed using high-fidelity enzyme Kapa Hifi HotStart. Size and quality of amplicons was evaluated in agarose gels and quantified with a Qubit 4.0 fluorometer and the Qubit dsDNA HS Assay Kit. Amplicons were then purified with magnetic pearls Mag-Bind® TotalPure NGS to eliminate non-specific products and primer dimers. A second PCR reaction was subsequently conducted with Platinum SuperFi II Polymerase HotStart ReadyMix enzyme to ligate Illumina sequencing adapters P5 and P7 attached to Illumina Nextera XT Index Kit v2 indices. Final libraries were purified with magnetic pearls Mag-Bind® TotalPure NGS and eluted into 22 μL 10 mM Tris. Fragment sizes was verified by electrophoresis capillary fragment analysis with kit DNF-910. Libraries for sequencing were quantified by fluorometry and diluted to 10 (Bacteria) or 4 nM (Archaea) DNA, pooled, denatured with 0.2 M NaOH for 5 min, and re-diluted to 750 (Bacteria) or 13 pM (Archaea). The PhiX control library was added (20%). Libraries were pooled and subjected to a 2 × 300 (forward and reverse) cycles Illumina paired end run with FastQ format.

The resulting fastq files were imported into Quime2 (Bolyen et al., 2019) and primer and adapter sequences removed. DADA2 was used to filter phiX reads, remove chimeras, and generate amplicon sequence variants (ASV). Multiple sequences were aligned, phylogenetic trees constructed, and taxonomies assigned using a Silva 138 SSU Ref NR99 database (Quast et al., 2012). Bacterial and archaeal diversity was calculated as total ASV, Faith’s Phylogenetic Diversity index, and Shannon index. Only those microbial clades whose relative abundance in at least one substrate by dilution rate combination and in at least one incubation and transfer, was equal or greater than 0.5%, were considered for the analysis of the relative abundance of bacterial and archaeal groups.

### 2.6 Calculations

#### 2.6.1 Dilution rate

Average dilution rates were calculated as:


$$\begin{array}{l} D\left(h^{-1}\right)=\frac{\text{total}\;\text{incubation}\;\text{volume}\;\left(\text{mL}\right)}{\text{inoculum}\;\left(\text{mL}\right)\times 72\;h}\\ \;=\frac{\left[\text{inoculum}\;\left(\text{mL}\right)+\text{fresh}\;\text{medium}\left(\text{mL}\right)\right]}{\text{inoculum}\;\left(\text{mL}\right)\times 72\;h} \end{array}$$

Therefore, the average dilution rates of serial cultures inoculated with 1, 2, or 4 mL were 0.56, 0.28, and 0.14 h^−1^, or 13.2, 6.6, or 3.3 d^−1^, respectively.

#### 2.6.2 Dry matter disappearance

Apparent dry matter disappearance (DMD) was calculated by subtracting the mass of the lyophilized dried residues from the dry matter mass of the substrate incubated and expressing the difference as a percentage.

#### 2.6.3 Gas production and composition

Gas pressure was calculated as the sum of gauge pressure plus 1 atm (101,325 Pa). The total number of moles contained in each bottle headspace was calculated applying the ideal gas law for the 60-mL gas volume contained in the bottles headspace. Production of CH_4_ and accumulation of H_2_ was calculated by multiplying their proportions in total gas by total gas amount. Dihydrogen partial pressure was calculated by multiplying total gas pressure by the proportion of H_2_.

#### 2.6.4 Reducing potential

Reducing potential is reported with the Standard Hydrogen Electrode (SHE) as a reference, by adding 197 mv to the E*h* values originally recorded (Ungerfeld et al., 2020).

#### 2.6.5 Net production of VFA

Production of VFA per bottle in each incubation transfer was calculated considering the volume of inoculum received and the concentration of each VFA in the donor bottle of the previous transfer (or in the original inoculum in case of transfer 1), and the final VFA concentration in the bottle in question.

#### 2.6.6 Bacteria and methanogens average doubling time

The total copies of the 16S rRNA gene and *mcrA* of bacteria and methanogens, respectively, initially inoculated into each transfer 1 bottle were calculated from the copies of 16S rRNA gene and *mcrA* copies per gram of inocula solids, the solids content per milliliter of inocula, and the volume of inoculum delivered per bottle. The total copies of 16S rRNA gene and *mcrA* per bottle in incubation 1, transfer 8, and incubation 2, transfers 4 and 8, was calculated from the 16S rRNA gene and *mcrA* copies per gram of incubation residue and the dry mass of incubation residue after lyophilizing. The following relationship between the final and the initial 16S rRNA and *mcrA* copies was applied to calculate a constant, average replication rate:


$$Nit=Ni0\times {\left(1+R-D\right)}^{t\times 72}$$

Where *N_iT_* are total 16S rRNA gene or *mcrA* copies per bottle at the end of transfer *t* of incubation *i*, *N_i0_* are total 16S rRNA gene or *mcrA* copies inoculated at the beginning of transfer 1 in the bottle corresponding to the same sequence of serial transfers, *R* is the replication rate in h^−1^, *D* is dilution rate of the treatment in question in h^−1^, and *t* corresponds to transfer 8 in incubation 1 or transfers 4 or 8 in incubation 2, multiplied by the 72 h growth interval between successive inoculations. Solving for *R* results in:


R=NtN01t×72+D−1

Finally, doubling time in hours was calculated as the reciprocal of replication rate:


$$t=R^{-1}$$

#### 2.6.7 Isotopic enrichment of volatile fatty acids

Isotopic enrichment of acetate was determined based on the absolute intensity of *m*/*z* molecular ion peaks 117, 118, and 119, corresponding to the M + 0, M + 1, and M + 2 acetate isotopologs, respectively (Supplementary Figures 1A–F). Similarly, relative intensity of peaks corresponding to molecular ions *m*/*z* 131, 132, and 133 were selected to identify propionate isotopologs M + 0, M + 1, and M + 2, respectively, and molecular ions *m*/*z* 145, 146, and 147, were selected to identify butyrate isotopologs M + 0, M + 1, and M + 2, respectively.

Volatile fatty acids ^13^C isotope ratio (IR) was calculated as (Gross, 2017):


$$IRi=\frac{\left[\left(M+1\right)AIi+2\times \left(M+2\right)AIi\right]}{\left[2\times \left(M+0\right)AIi+\left(M+1\right)AIi\right]}$$

Where *IR_i_* is the ^13^C/^12^C isotope ratio of VFA *i* (i.e., acetate, propionate, or butyrate), and *AI_i_* are the absolute intensities of the M + 0, M + 1, and M + 2 isotopic peaks, each of them multiplied by their number of ^13^C carbons (numerator) or ^12^C carbons (denominator). The M + 3 propionate isotopolog, and M + 3 and M + 4 butyrate isotopologs, were not detected and hence were not considered in the calculations.

The isotopic enrichment of each VFA in each incubation bottle relative to the bottle of the same treatment that had received unlabeled ^13^C medium as a natural abundance standard (see section 2.3) was calculated and reported using the delta notation (Gross, 2017):

δ^13^C‰*ij* = ($\frac{\text{IRij}\;\text{labeled}}{\text{IRij}\;std}-1)\times 1000.$ Where δ^13^C‰*ij* is the ^13^C delta enrichment of sample *i* in VFA *j* (i.e., acetate, propionate, or butyrate).

### 2.7 Statistical analyses

There were three different incubation bottles per combination of type of substrate and dilution rate inoculated in transfer 1, which gave rise each to three different sequences of serially transferred cultures per combination of type of substrate and dilution rate. The statistical model included the main fixed effects of type of substrate (S), dilution rate (D), and transfer number (T), and the random effects of incubation (I) and sequence nested in the incubation, and the three double interactions between S, D, and T, and their triple interaction. Preliminary analyses revealed important differences between the first and the second serial incubations in the evolution of key response variables throughout the eight transferred cultures depending on type of substrate and dilution rate. The random triple interactions between the incubation, type of substrate, and transfer number (I × S × T), and incubation, dilution rate, and transfer number (I × D × T) were generally significant (*p* < 0.05) or tendencies (0.05 ≤ *p* < 0.10) and were therefore included in the model. The statistical model used thus was:


$$\begin{array}{l} \text{response}=\text{overall}\;\text{mean}+\text{substrate}\;\left(S\right)+\text{dilution}\;\text{rate}\left(D\right)\\ +\text{transfer}\;\text{number}\left(T\right)+\left(S\times D\right)+\left(S\times T\right)+\left(D\times T\right)\\ +\left(S\times D\times T\right)+\text{sequence}\;\left(\text{incubation},\;\text{random}\right)\\ +\text{incubation}\;\left(I,\;\text{random}\right)+\left(I\times S\times T,\;\text{random}\right)\\ +\left(I\times D\times T,\;\text{random}\right)+\text{error} \end{array}$$

Apparent DM digestibility and microbial abundance and community composition, which were analyzed in transfer 8 of incubation 1, and transfers 4 and 8 of incubation 2, and ^13^C enrichment in acetate, propionate, and butyrate, which was analyzed only in transfer 8 of incubation 2, were separately analyzed per incubation and transfer with the following model:


$$\begin{array}{l} \text{response}=\text{overall}\;\text{mean}+\text{substrate}+\text{dilution}\;\text{rate}\\ \;+\left(\text{substrate}\times \text{dilution}\;\text{rate}\right)+\text{error} \end{array}$$

The null hypothesis of ^13^C enrichment in acetate, propionate, and butyrate being equal to zero was firstly examined for the overall means of all combination treatments of substrate by dilution rate.

Significance was considered at *p* < 0.05 and tendencies at 0.05 ≤ *p* < 0.10. When the main effect of dilution rate was significant, linear responses to dilution rate were evaluated through polynomial contrasts for the unequal levels of dilution rate used (5, −1, and − 4 for low, medium, and high dilution rate, respectively). If interactions between type of substrate and dilution rate occurred (*p* < 0.05), the high forage and high concentrate substrate were compared within each dilution rate level separately.

Outliers were identified as observations falling outside of the 99.9% central distribution of studentized residuals. Outliers so identified were first examined for evident measurement or typing problems. Outliers that clustered with other outliers of the same combination of substrate and dilution rate treatments and belonged to the same transfer or to consecutive transfers, or outliers that belonged to the same sequence and clustered together, were considered biological results and were kept in the analyses. In other cases, the analysis was conducted again with each outlier removed at a time. Other outliers were only eliminated from an analysis if the central conclusions of the analysis changed after eliminating them. Finally, none of the outliers was found to be influential, and thus all observations were retained for all response variables.

In addition, Pearson correlations were conducted between CH_4_, acetate, propionate, and butyrate production, and lactate concentration, with H_2_ partial pressure, and between H_2_ accumulation and pH. A principal component analyses of fermentation variables and bacterial phyla, and a hierarchical cluster analysis of bacterial genera, were also conducted. All statistical analyses were conducted with JMP 17.2.0 (JMP®, 2023).

## 3 Results

### 3.1 Digestion and fermentation

Except for greater DMD of the high-concentrate substrate in incubation 1, transfer 8 (*p* = 0.014; Supplementary Table S5), there were no effects of substrate (*p* ≥ 0.15), dilution rate (*p* ≥ 0.20), or their interaction (*p* ≥ 0.27) on DMD. In transfer 8 of both incubations, DMD was negative (incubation 1) or close to zero (incubation 2), denoting greater or similar microbial biomass accretion than disappearance of incubated substrate, respectively.

**Figure 2:**
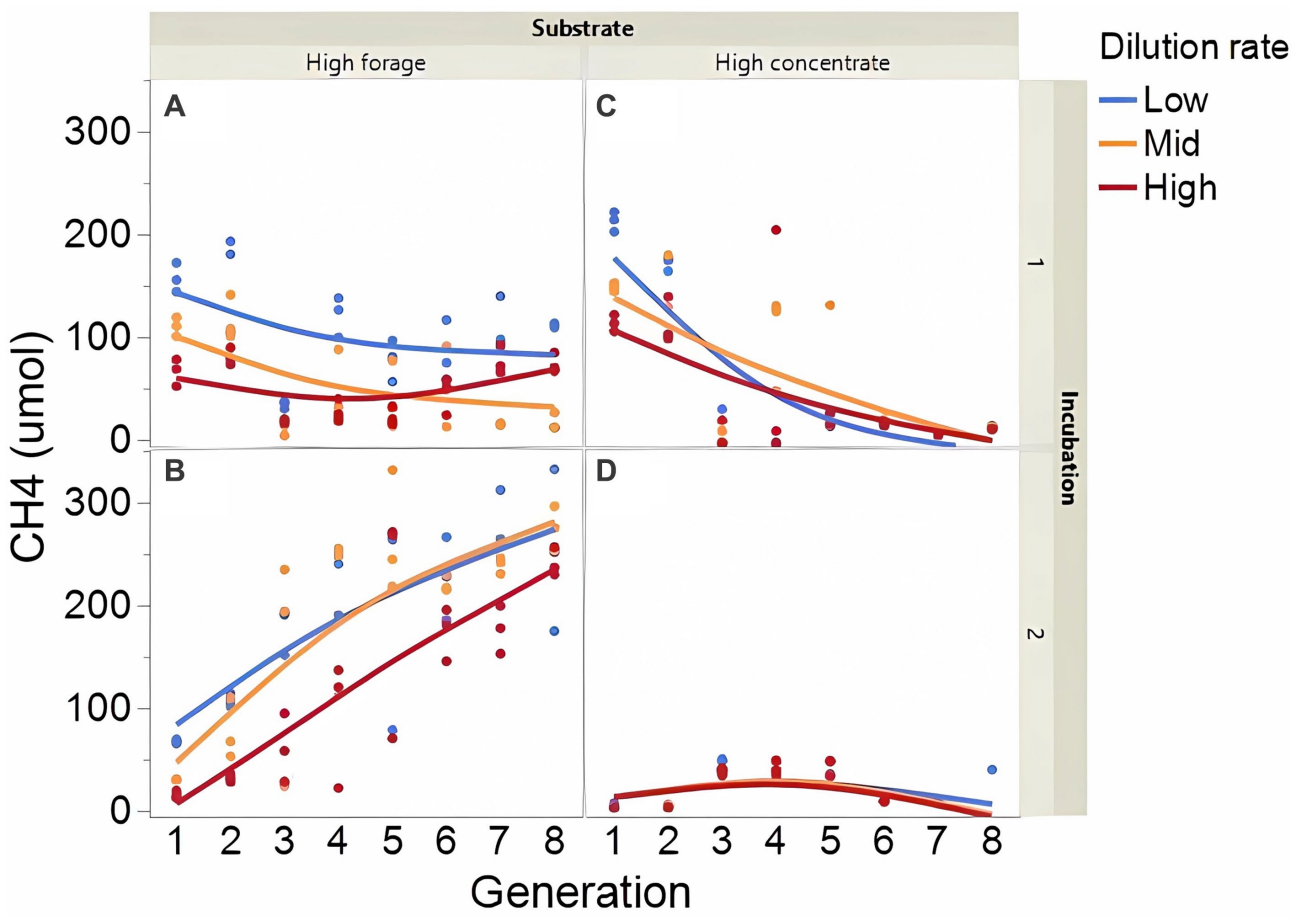
Evolution of methane (CH_4_) production in 8 transfers of serial rumen cultures growing on a high forage **(A,B)** or a high concentrate **(C,D)** substrate, at three average dilution rates. The experiment was conducted twice: Incubation 1 **(A,C)** and 2 **(B,D)**. Substrate (S): *p* = 0.008; Dilution rate (D): *p* = 0.003; Transfer (T): *p* = 0.99; (S × D): *p* = 0.010; (S × T): *p* = 0.74; (D × T): *p* = 0.44; (S × D × T): *p* = 0.014; Incubation (I, random): *p* = 0.90; (I × S × T, random): *p* = 0.009; (I × D × T, random): *p* = 0.26; Sequence [Incubation, random] *p* = 0.012.

**Figure 3:**
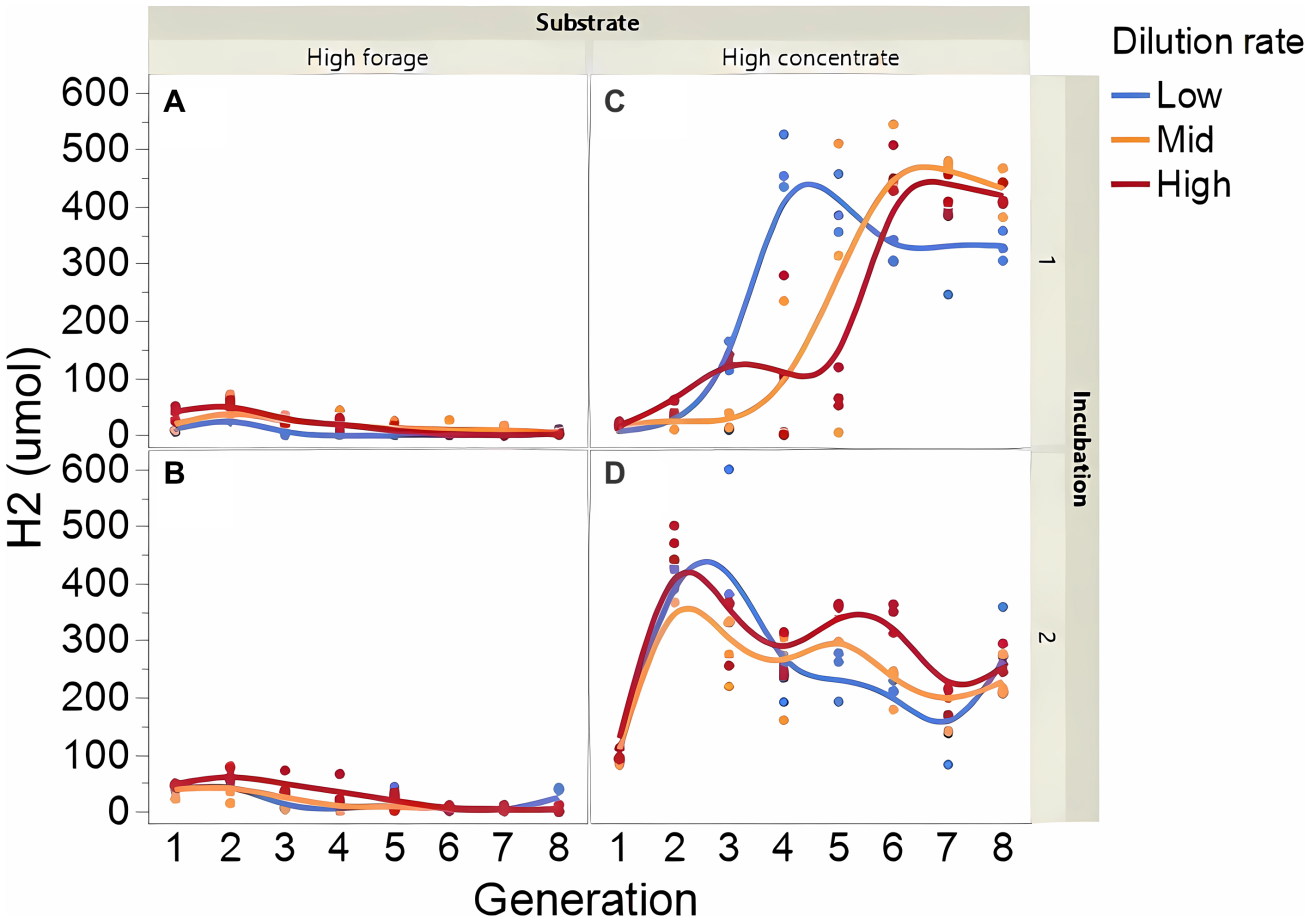
Evolution of dihydrogen (H_2_) accumulation in 8 transfers of serial rumen cultures growing on a high forage **(A,B)** or a high concentrate **(C,D)** substrate, at three dilution rates. The experiment was conducted twice: Incubation 1 **(A,C)** and 2 **(B,D)**. Substrate (S): *p* < 0.001; Dilution rate (D): *p* = 0.80; Transfer (T): *p* = 0.75; (S × D): *p* = 0.44; (S × T): *p* = 0.49; (D × T): *p* = 0.57; (S × D × T): *p* = 0.014; Incubation (I, random): *p* = 0.23; (I × S × T, random): *p* = 0.011; (I × D × T, random): *p* = 0.037; Sequence [Incubation, random]: *p* = 0.38.

**Figure 4:**
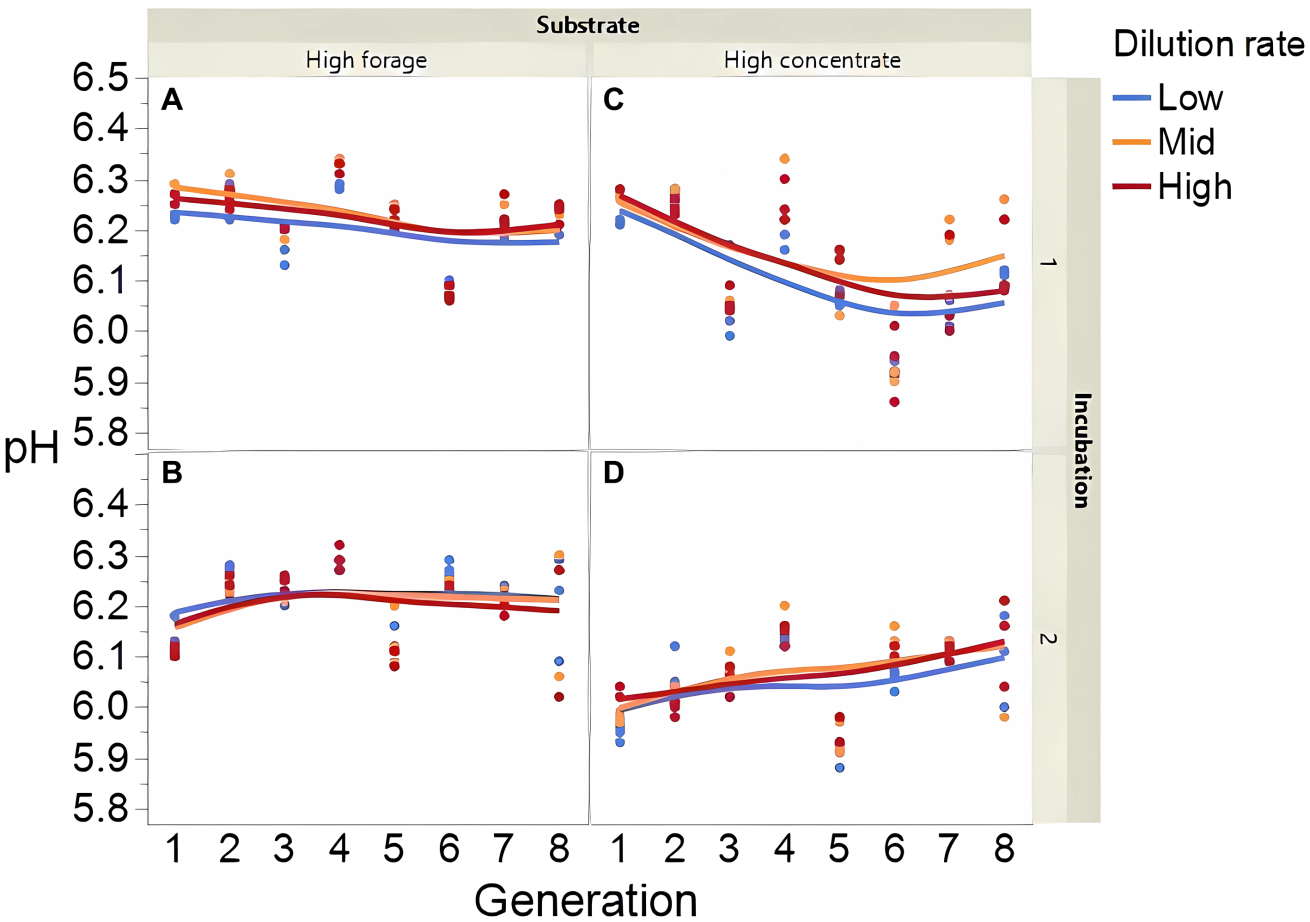
Evolution of pH in 8 transfers of serial rumen cultures growing on a high forage **(A,B)** or a high concentrate **(C,D)** substrate, at three dilution rates. The experiment was conducted twice: Incubation 1 **(A,C)** and 2 **(B,D)**. Substrate (S): *p* = 0.001; Dilution rate (D): *p* = 0.024; Transfer (T): *p* = 0.24; (S × D): *p* = 0.29; (S × T): *p* = 0.99; (D × T): *p* = 0.042; (S × D × T): *p* = 0.14; Incubation (I, random): *p* = 0.74; (I × S × T, random): *p* = 0.007; (I × D × T, random): *p* = 0.016; Sequence [Incubation, random]: *p* = 0.028.

Fermentation results varied amply between both incubation runs; thus, figures showing each incubation run are presented separately. The high concentrate substrate produced more total gas than the high forage substrate (*p* < 0.001; Supplementary Figure 2), especially in incubation 1 and as the incubation progressed (I × S × T: *p* = 0.010). Total gas production linearly decreased as dilution rate increased (*p* < 0.001). Methane production steadily increased with high forage in incubation 2 but not in incubation 1 as the incubation progressed and decreased or remained low with high concentrate (Figure 2; I × S × T: *p* = 0.009). Methane production linearly decreased with dilution rate with high forage but was unaffected with high concentrate (S × D: *p* = 0.010). Accumulation of H_2_ (net production of H_2_) was on average 14.5-fold greater with high concentrate (*p* < 0.001; Figure 3) and increased throughout the high concentrate serial incubations, especially in incubation 1, while it decreased with high forage as the serial incubations progressed (I × S × T: *p* = 0.011). High concentrate incubations had on average a lower 72-h pH than their high forage counterparts (*p* = 0.002; Figure 4). At low, but not at mid or high dilution rates, high forage incubations had a lower 72-h E*h* compared to high concentrate (S × D: *p* < 0.001; Supplementary Figure S3). Ammonium concentration increased throughout the incubations with both substrates in incubation 1 and decreased after transfer 3 with high concentrate in incubation 2 (I × S × T: *p* = 0.019; Supplementary Figure S4).

**Figure 5:**
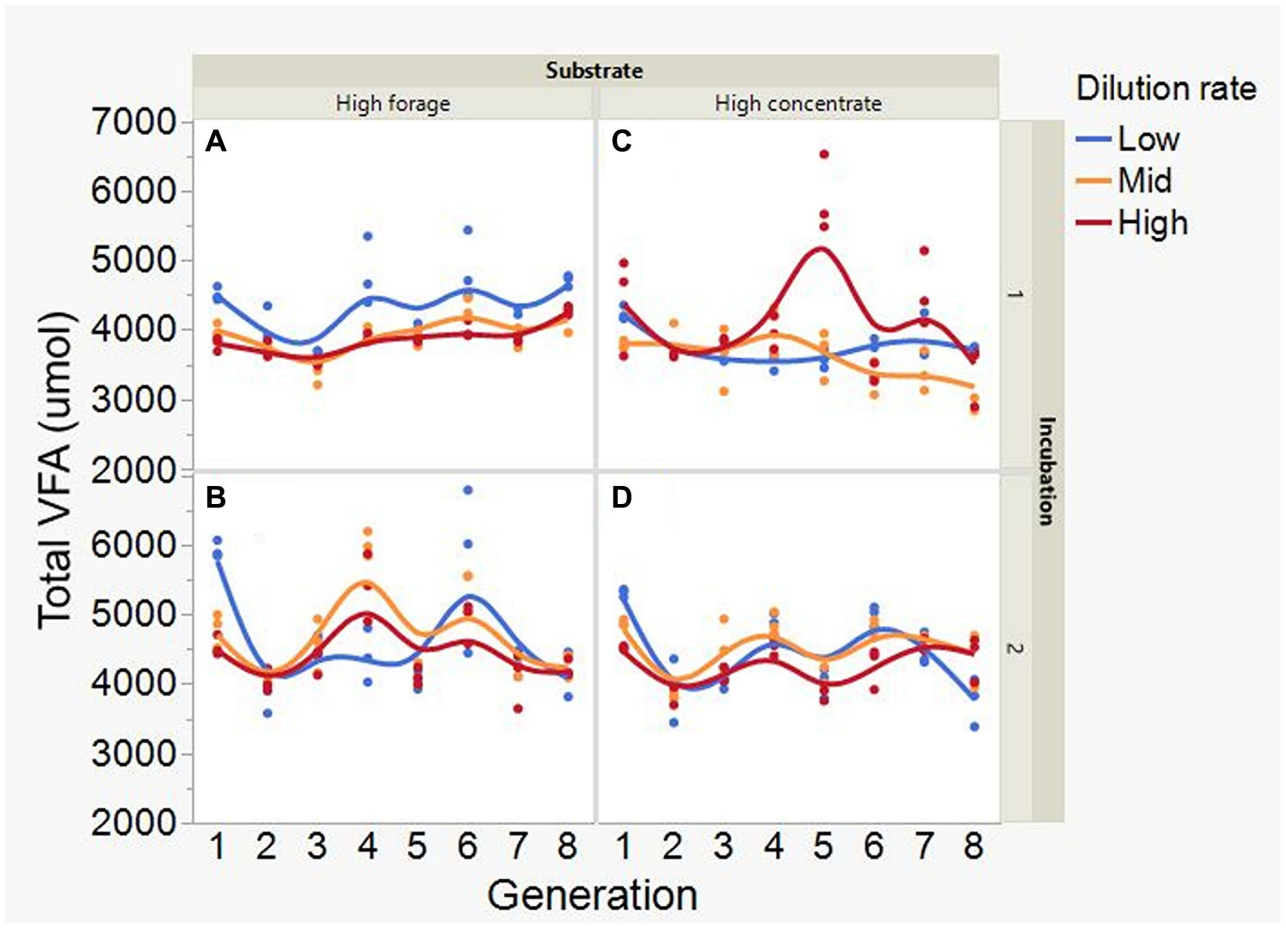
Evolution of total VFA concentration in 8 transfers of serial rumen cultures growing on a high forage **(A,B)** or a high concentrate **(C,D)** substrate, at three dilution rates. The experiment was conducted twice: Incubation 1 **(A,C)** and 2 **(B,D)**. Substrate (S): *p* = 0.071; Dilution rate: (D) *p* = 0.57; Transfer (T): *p* = 0.053; (S × D): *p* = 0.004; (S × T): *p* = 0.27; (D × T): *p* = 0.38; (S × D × T): *p* = 0.11; Incubation (I, random): *p* = 0.50; (I × S × T, random): *p* = 0.099; (I × D × T, random): *p* = 0.039; Sequence [Incubation, random]: *p* = 0.20.

**Figure 6:**
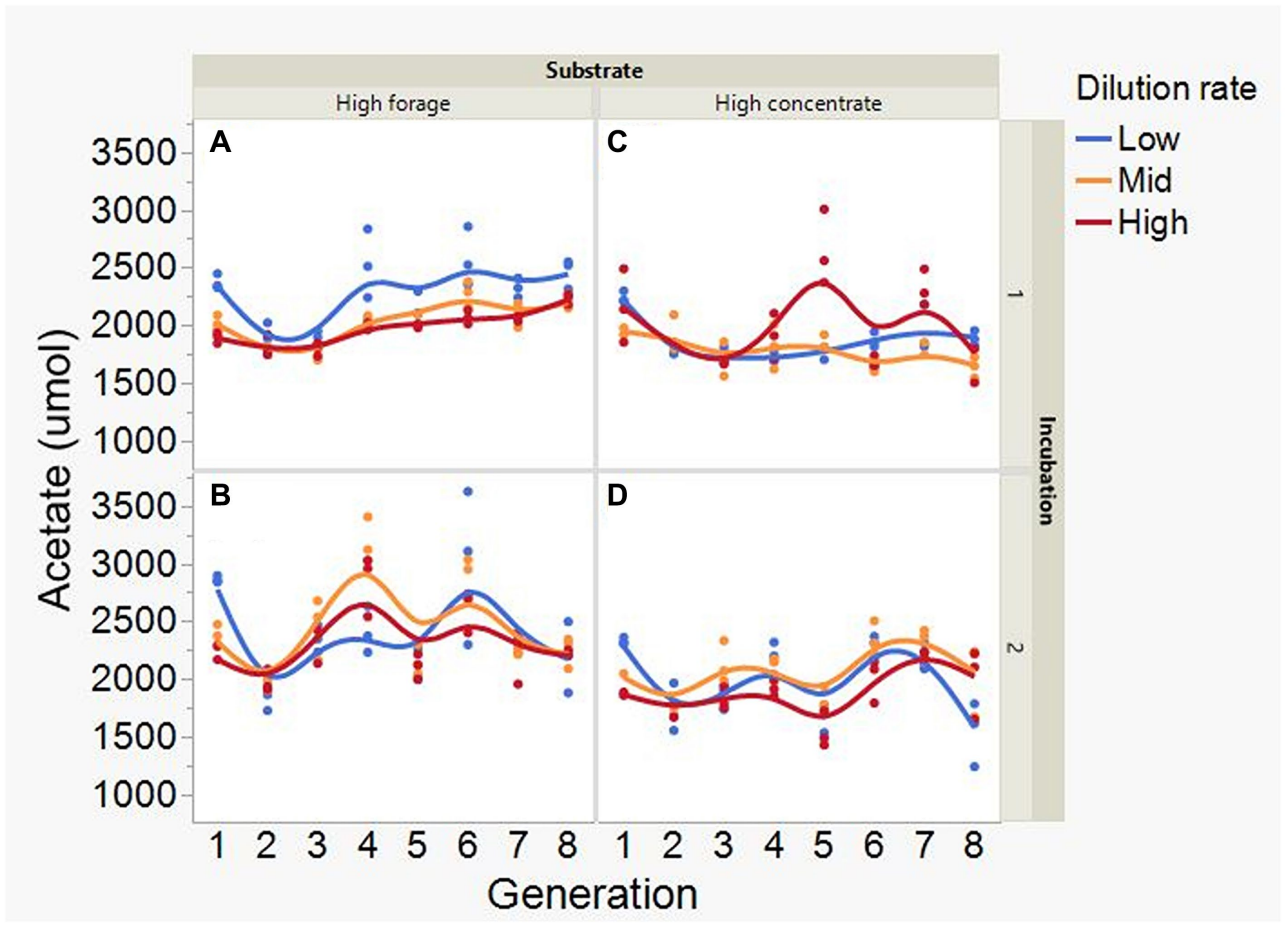
Evolution of acetate production in 8 transfers of serial rumen cultures growing on a high forage **(A,B)** or a high concentrate **(C,D)** substrate, at three dilution rates. The experiment was conducted twice: Incubation 1 **(A,C)** and 2 **(B,D)**. Substrate (S): *p* < 0.001; Dilution rate (D): *p* = 0.34; Transfer (T): *p* = 0.073; (S × D): *p* = 0.025; (S × T): *p* = 0.31; (D × T): *p* = 0.31; (S × D × T): *p* = 0.38; Incubation (I, random): *p* = 0.55; (I × S × T, random): *p* = 0.044; (I × D × T, random): *p* = 0.084; Sequence [Incubation, random]: *p* = 0.12.

**Figure 7:**
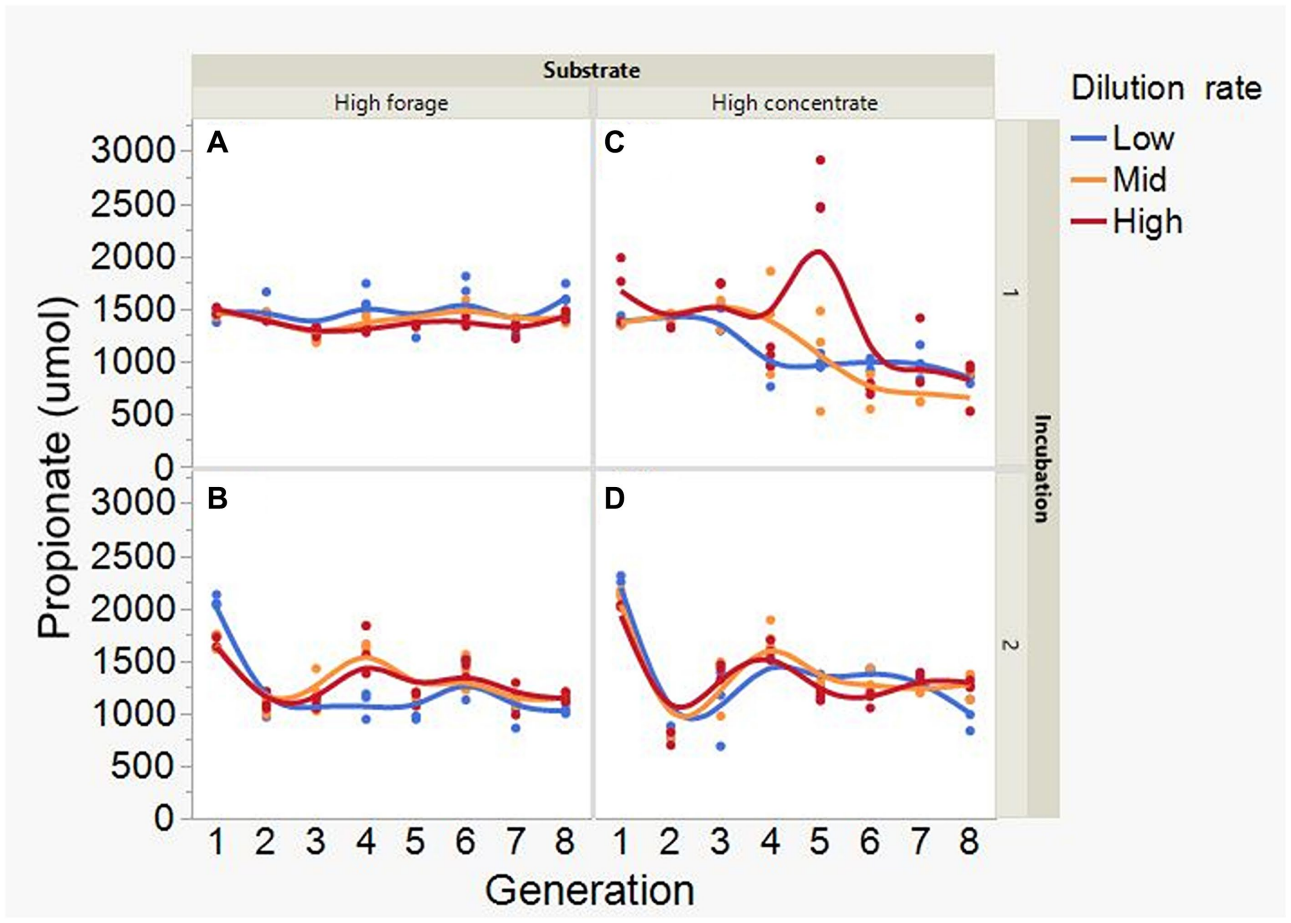
Evolution of propionate production in 8 transfers of serial rumen cultures growing on a high forage **(A,B)** or a high concentrate **(C,D)** substrate, at three dilution rates. The experiment was conducted twice: Incubation 1 **(A,C)** and 2 **(B,D)**. Substrate (S): *p* = 0.48; Dilution rate (D): *p* = 0.61; Transfer (T): *p* = 0.15; (S × D): *p* = 0.068; (S × T): *p* = 0.57; (D × T): *p* = 0.44; (S × D × T): *p* < 0.001; Incubation (I, random): *p* = 0.003; (I × S × T, random): *p* = 0.019; (I × D × T, random): *p* = 0.025; Sequence [Incubation, random]: *p* = 0.063.

**Figure 8:**
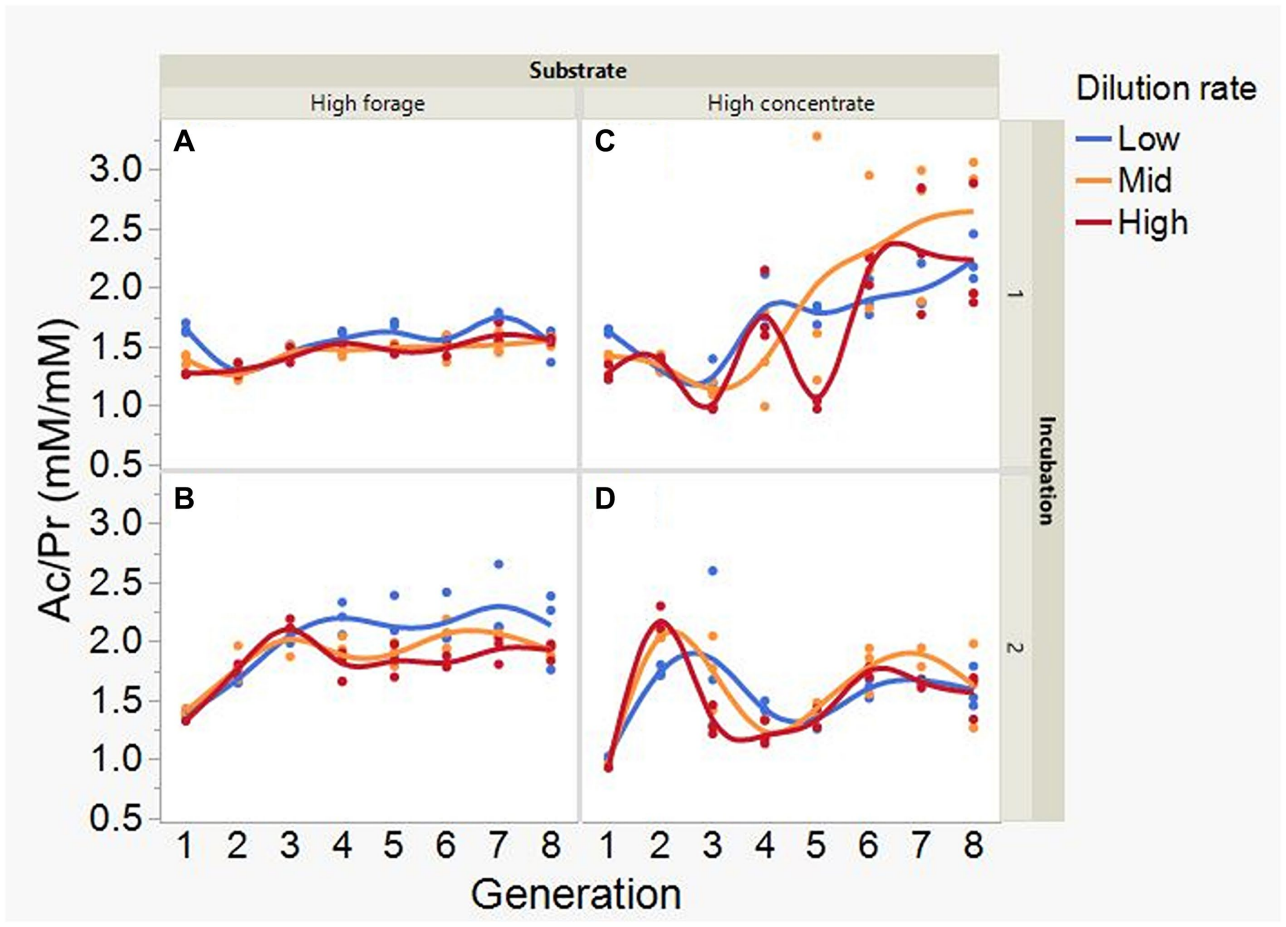
Evolution of the acetate to propionate molar ratio (Ac/Pr) in 8 transfers of serial rumen cultures growing on a high forage **(A,B)** or a high concentrate **(C,D)** substrate, at three dilution rates. The experiment was conducted twice: Incubation 1 **(A,C)** and 2 **(B,D)**. Substrate (S): *p* = 0.70; Dilution rate (D): *p* = 0.17; Transfer (T): *p* = 0.26; (S × D): *p* = 0.19; (S × T): *p* = 0.81; (D × T): *p* = 0.18; Incubation (I, random): *p* = 0.72; (I × S × T, random): *p* = 0.012; (I × D × T, random): *p* = 0.24; Sequence [Incubation, random]: *p* = 0.009.

**Figure 9:**
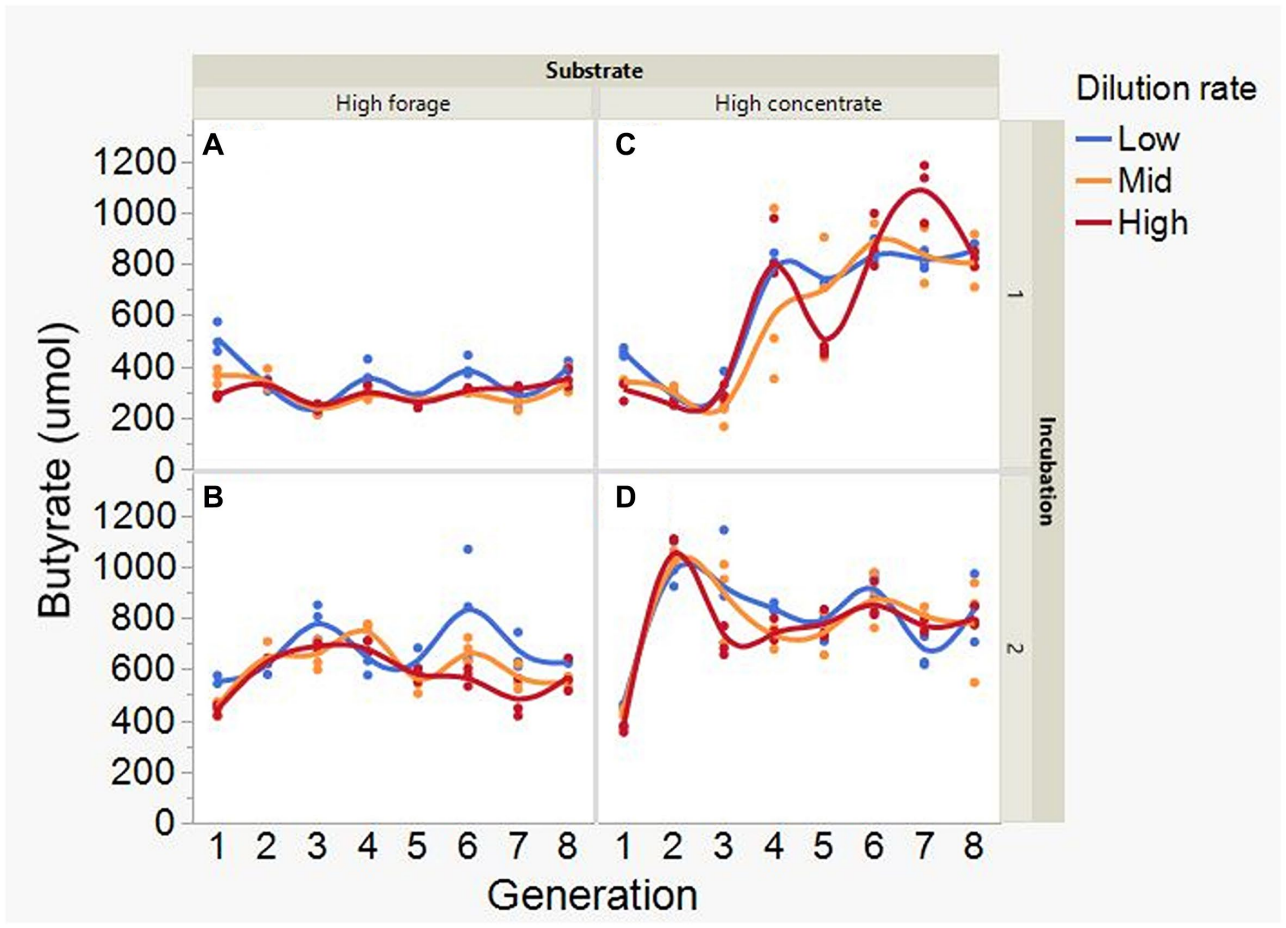
Evolution of butyrate production in 8 transfers of serial rumen cultures growing on a high forage **(A,B)** or a high concentrate **(C,D)** substrate, at three dilution rates. The experiment was conducted twice: Incubation 1 **(A,C)** and 2 **(B,D)**. Substrate (S): *p* = 0.001; Dilution rate (D): *p* = 0.040; Transfer (T): *p* = 0.40; (S × D): *p* = 0.25; (S × T): *p* = 0.59; (D × T): *p* = 0.57; Incubation (I, random): *p* = 0.51; (I × S × T, random): *p* = 0.009; (I × D × T, random): *p* = 0.072; Sequence [Incubation, random]: *p* = 0.55.

At low and mid, but not at high, dilution rate, the high forage incubations had (*p* = 0.005) or tended (*p* = 0.052) to have greater VFA production compared to high concentrate (S × D: *p* = 0.004; Figure 5). Acetate production was greater with the high forage substrate (*p* < 0.001), although the difference with the high concentrate substrate decreased with increasing dilution rate (S × D: *p* = 0.025; Figure 6). In incubation 1, propionate production decreased throughout the serial incubations with high concentrate, but not with high forage, while in incubation 2 the evolution of propionate production was similar for the high forage and the high concentrate substrates (I × S × T: *p* = 0.019; Figure 7). On average, there was no effect of substrate (*p* = 0.70) or dilution rate (*p* = 0.17) on the acetate to propionate molar ratio (Figure 8). In incubation 1, with the high concentrate, but not with the high forage substrate, there was an increase in the acetate to propionate molar ratio as the incubation progressed. The acetate to propionate molar ratio increased at the beginning of incubation 2 with the high forage substrate and plateaued in transfer 3, while with the high concentrate substrate it peaked in transfer 2 and oscillated thereafter (I × S × T: *p* = 0.012; Figure 8). With the high concentrate substrate, butyrate production sharply increased beginning in transfer 4 of incubation 1 and peaked in transfer 2 in incubation 2, declining slightly thereafter. With the high forage substate, butyrate production changed little throughout the incubations (I × S × T: *p* = 0.009; Figure 9). Butyrate production linearly decreased with increasing dilution rate (*p* = 0.040).

Isobutyrate production was greater with high forage at the mid dilution rate (*p* = 0.048) and tended (*p* = 0.087) to be greater at the low dilution rate (S × D: *p* = 0.010; Supplementary Figure S5), although this was affected by transfer number (S × D × T: *p* = 0.002). Production of 2- and 3-methylbutyrate changed little throughout incubation 1 with both substrates and decreased and increased slightly with the high forage and the high concentrate substrates, respectively, in incubation 2 (I × S × T: *p* = 0.021; Supplementary Figure S6). In incubation 1, valerate production increased in the first three transfers with the high forage substrate, and it decreased with the high concentrate substrate. In incubation 2, valerate production peaked in transfer 6 with the high forage substrate, and generally increased as the incubation progressed with the high concentrate substrate (I × S × T: *p* = 0.013; Supplementary Figure S7). Production of 4-methylvalerate peaked in transfer 3 in incubation 1 with both substrates, albeit to a greater level with high forage, and decreased in incubation 2 as the incubation progressed (I × S × T: *p* = 0.008; Supplementary Figure S8). Caproate production was greater with high forage (*p* < 0.001; Supplementary Figure S9), with the differences becoming smaller as dilution rate increased (S × D: *p* = 0.034). Production of heptanoate was greater with the high forage substrate and with the low dilution rate in transfer 1 of incubation 2 (I × S × T: *p* = 0.026 and I × D × T: *p* = 0.016; Supplementary Figure S10).

**Figure 10:**
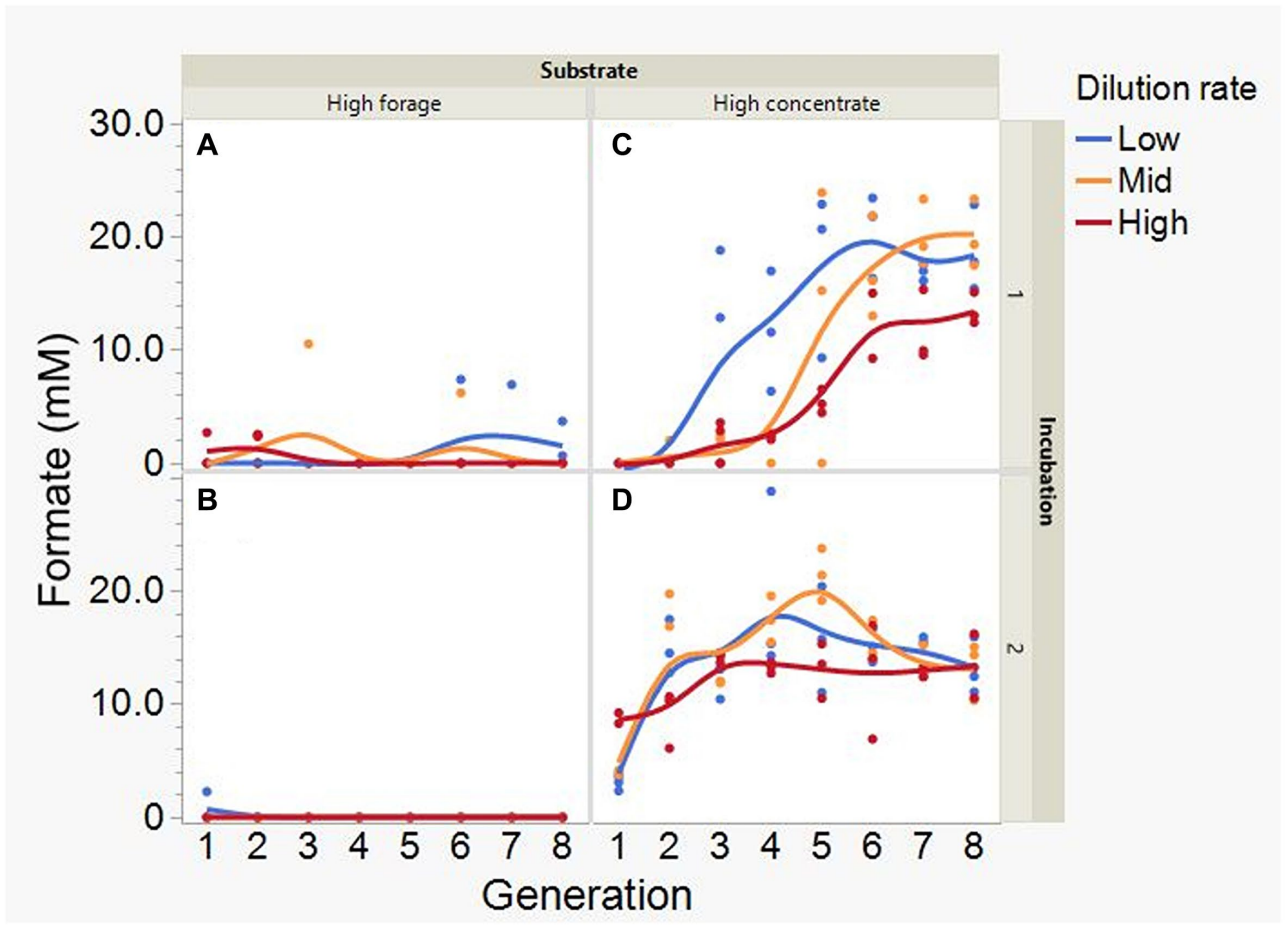
Evolution of formate accumulation in 8 transfers of serial rumen cultures growing on a high forage **(A,B)** or a high concentrate **(C,D)** substrate, at three dilution rates. The experiment was conducted twice: Incubation 1 **(A,C)** and 2 **(B,D)**. Substrate (S): *p* < 0.001; Dilution rate (D): *p* = 0.008; Transfer (T): *p* = 0.18; (S × D): *p* = 0.009; (S × T): *p* = 0.18; (D × T): *p* = 0.36; (S × D × T): *p* = 0.007; Incubation (I, random): *p* = 0.75; (I × S × T, random): *p* = 0.012; (I × D × T, random): *p* = 0.37; Sequence [Incubation, random]: *p* = 0.15.

**Figure 11:**
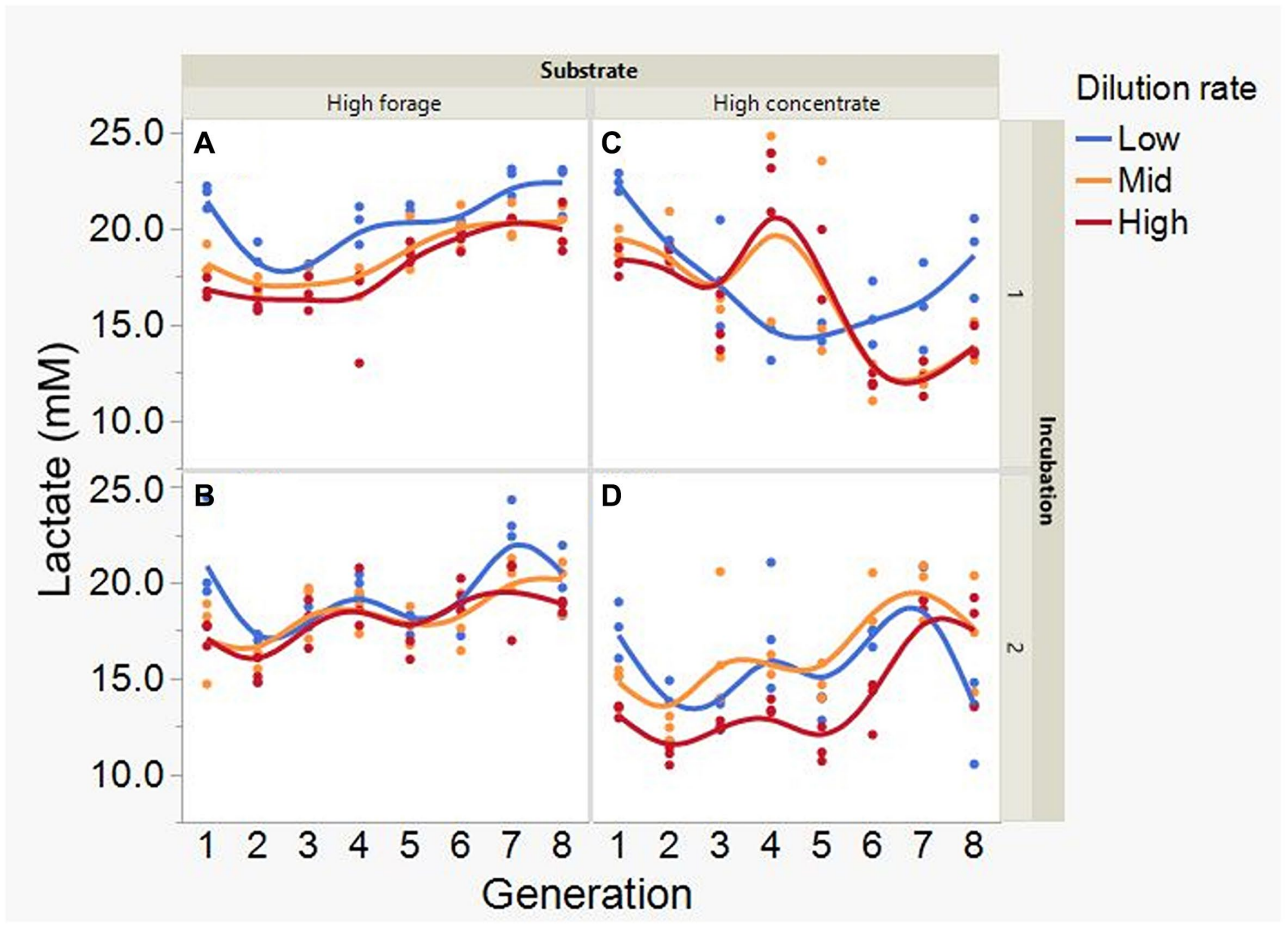
Evolution of lactate accumulation in 8 transfers of serial rumen cultures growing on a high forage **(A,B)** or a high concentrate **(C,D)** substrate, at three dilution rates. The experiment was conducted twice: Incubation 1 **(A,C)** and 2 **(B,D)**. Substrate (S): *p* = 0.002; Dilution rate (D): *p* = 0.007; Transfer (T): *p* = 0.60; (S × D): *p* = 0.11; (S × T): *p* = 0.73; (D × T): *p* = 0.57; (S × D × T): *p* = 0.068; Incubation (I, random): *p* = 0.90; (I × S × T, random): *p* = 0.016; (I × D × T, random): *p* = 0.077; Sequence [Incubation, random]: *p* = 0.32.

Average formate accumulation was 34-fold greater with the high concentrate substrate (*p* < 0.001; Figure 10), and increased with high concentrate as the incubations progressed, plateauing at transfer 6 in incubation 1 and peaking at transfer 4 or 5, depending on dilution rate, in incubation 2 (I × S × T: *p* = 0.012). Formate accumulation linearly decreased with increasing dilution rate with high concentrate (*p* < 0.001; S × D: *p* = 0.009). Lactate accumulation was on average greater with high forage (*p* = 0.002) and linearly decreased with dilution rate (*p* = 0.003; Figure 11). Lactate gradually increased with high forage in incubations 1 and 2. With high concentrate, lactate decreased in incubation 1 until transfer 6, and in incubation 2 it reached a minimum in transfer 2 and increased thereafter (I × S × T: *p* = 0.016). In incubation 1, succinate decreased with both substrates, while in incubation 2, succinate decreased only with the high forage substrate and did not accumulate with high concentrate (I × S × T: *p* = 0.037; Supplementary Figure S11).

**Figure 12:**
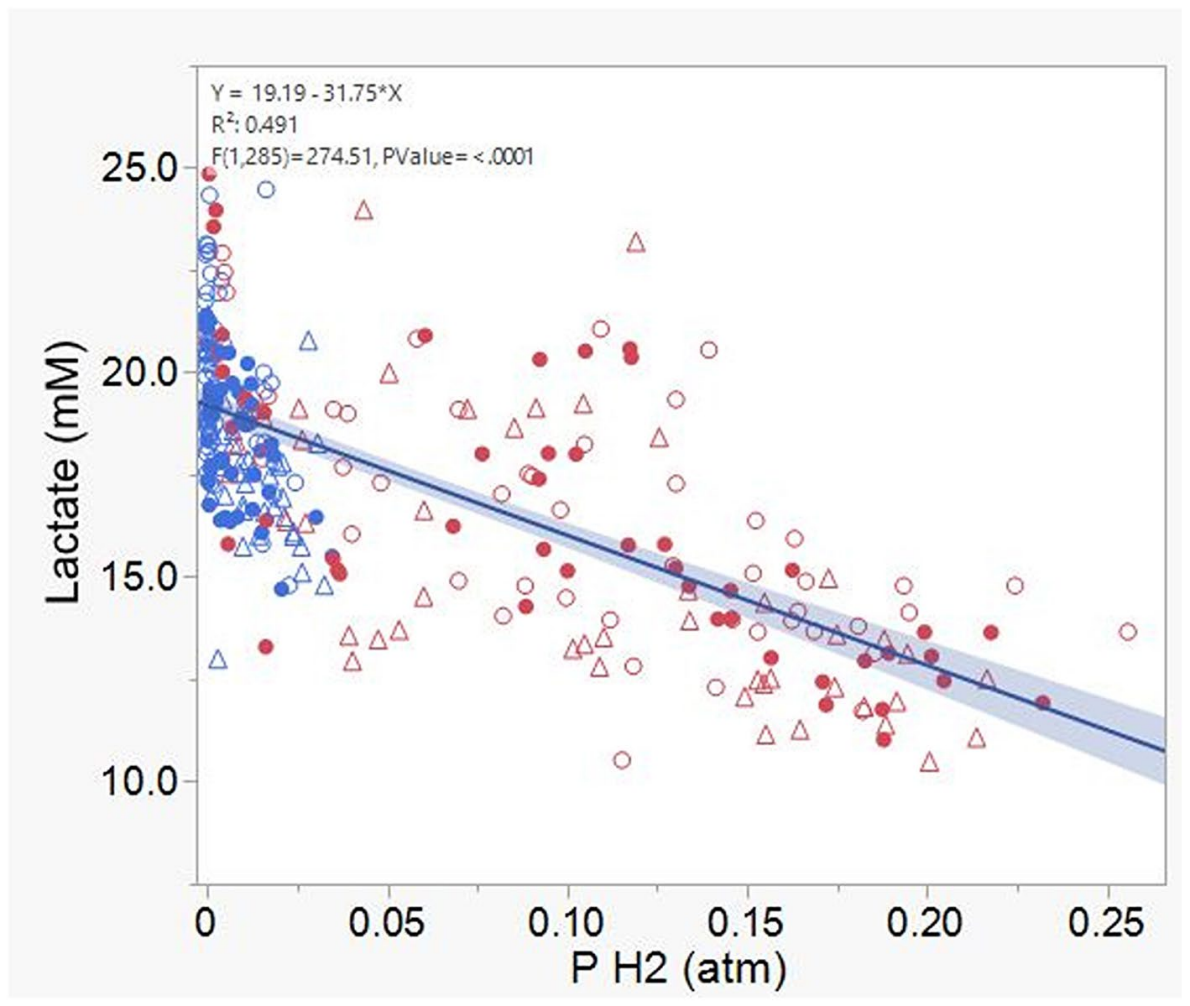
Relationship between lactate concentration and dihydrogen (H_2_) partial pressure in 8 transfers of serial rumen cultures growing on a high forage and or a high concentrate substrate, at three dilution rates. Blue symbols, high forage; Red symbols, high concentrate; Hollow circles, low dilution rate; Solid circles, mid dilution rate; Triangles, high dilution rate. Shaded area indicates the 95% confidence band.

**Figure 13:**
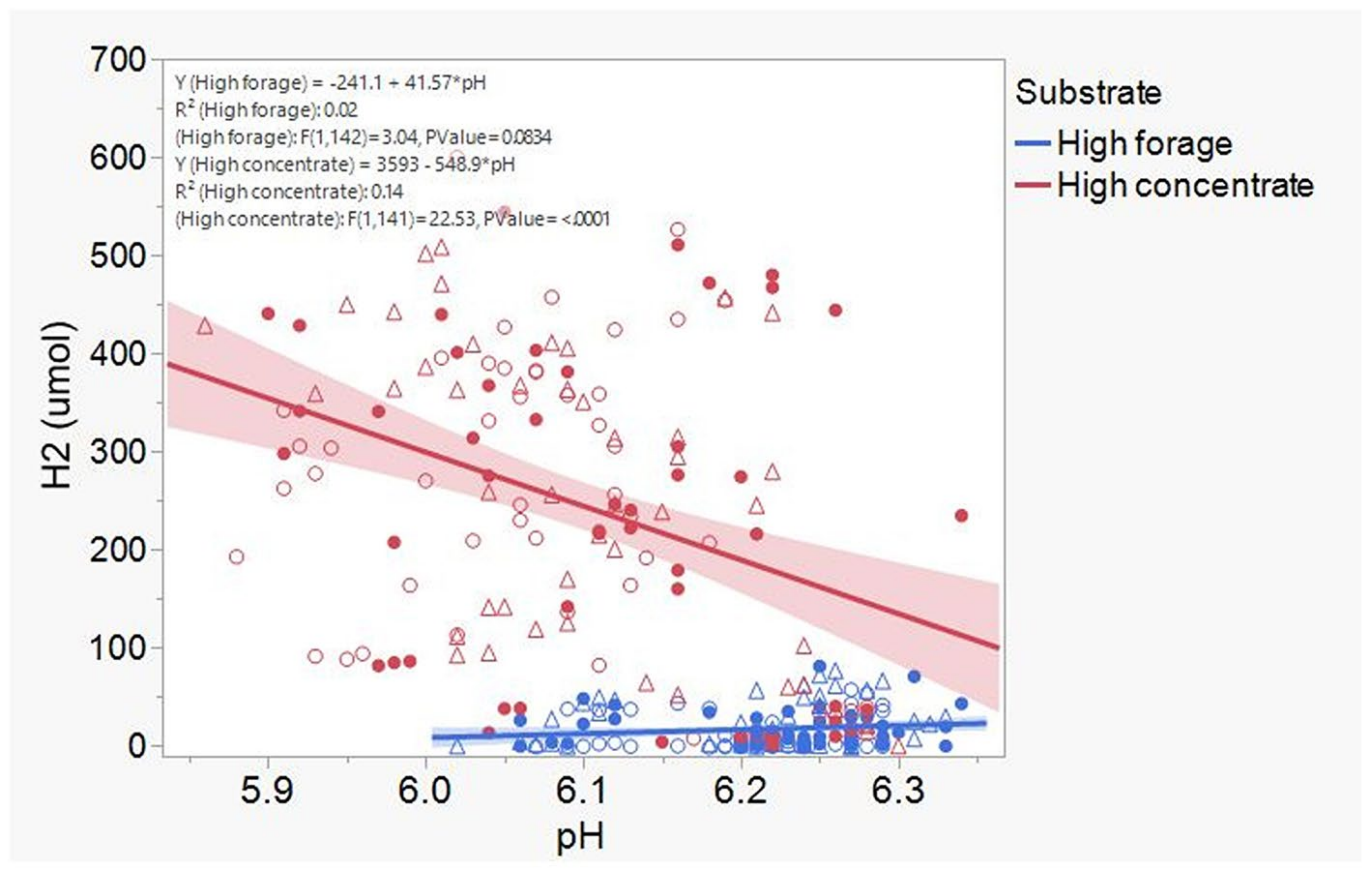
Relationship dihydrogen (H_2_) accumulation and culture final pH in 8 transfers of serial rumen cultures growing on a high forage and or a high concentrate substrate, at three dilution rates. Blue symbols, high forage; Red symbols, high concentrate; Hollow circles, low dilution rate; Solid circles, mid dilution rate; Triangles, high dilution rate. Shaded area indicates the 95% confidence band.

Acetate (Supplementary Figure S12) and CH_4_ production (Supplementary Figure S13), and lactate concentration (Figure 12), all had a negative association with H_2_ partial pressure. Accumulation of H_2_ beyond 0.05 atm was also negatively associated with propionate production (Supplementary Figure S14). Conversely, H_2_ pressure was positively associated with butyrate production (Supplementary Figure S15). Dihydrogen accumulation was considerably greater with high concentrate than with high forage even at similar pH (Figure 13). A principal component analysis biplot of fermentation variables showed a clear separation between substrates in principal component 1, but not between dilution rates (Supplementary Figure S16). High concentrate associated the most with the accumulation of H_2_ and formate and total gas production, and high forage with CH_4_, acetate, propionate, isobutyrate, valerate, caproate, heptanoate, and total VFA production, pH, and lactate concentration. In incubation 1, the high forage and high concentrate substrates gradually separated as transfers progressed, while in incubation 2, they were already well separated since the onset of transfer 1 (results not shown).

### 3.2 Incorporation of bicarbonate carbon into volatile fatty acids

Overall delta values for acetate (*p* = 0.15) and propionate (*p* = 0.59) were not different from zero, however, butyrate was on average enriched in ^13^C (*p* = 0.008; results not shown). There were no effects of type of substrate or dilution rate on the ^13^C enrichment of acetate, propionate, or butyrate (*p* ≥ 0.22).

### 3.3 Absolute abundance of bacteria and archaea

In incubation 1, transfer 8, bacteria were more abundant with high forage at low and high, but not at mid, dilution rate (S × D: *p* = 0.016; Supplementary Table S6). In incubation 2, transfer 8, there was a tendency to greater (*p* = 0.058) bacterial abundance with high concentrate, while there were no differences in incubation 2, transfer 4 (*p* = 0.39). Archaeal abundance was always greater with high forage (*p* < 0.001) and little influenced by dilution rate (*p* ≥ 0.28). Bacterial 16S rRNA gene copies per gram of inoculum in the initial inocula from incubations 1 and 2 on a log_10_ basis were equal to 10.6 and 10.5, respectively. Archaeal 16S rRNA gene copies per gram of inoculum in the initial inocula from incubations 1 and 2 on a log_10_ base were equal to 7.80 and 6.81, respectively.

As expected, doubling times of bacteria and archaea decreased with increasing dilution rates (Supplementary Table S6). Archaeal replication rates were slightly higher than dilution rates with high forage, and slightly lower than dilution rates with high concentrate (results not shown), thus archaeal numbers increased and decreased with the high forage and high concentrate substrate, respectively.

### 3.4 Bacterial community composition

A total of 16,317,083 bacterial raw reads were sequenced, with an average of 291,376 reads per sample (288,201 reads for incubation residues and 377,119 for initial inocula), with a minimum of 215,272 and a maximum of 552,674. Total unique bacterial amplicon sequence variants, Faith phylogenetic diversity index, and Shannon diversity index, were consistently higher with high forage than with high concentrate substrate (*p* < 0.001; Supplementary Table S7). There were no effects of dilution rate on bacterial diversity variables (*p* ≥ 0.14). Total unique bacterial amplicon sequence variants, Faith phylogenetic diversity index, and Shannon diversity index were numerically lesser in the cultures than in their corresponding inocula (result not shown).

A principal component analysis biplot of bacterial phyla showed a clear separation between high forage and high concentrate substrates, with no clear influence of dilution rate (Supplementary Figure S17). Incubations with high forage generally had greater relative abundance of phyla Bacteroidota, Actinobacterota, and Verrucomicrobiota, and those with high concentrate generally had more Proteobacteria, Synergistota, Spirochaetota, and Desulfobacterota (Supplementary Table S8). Firmicutes had greater relative abundance with high forage, except for incubation 2, transfer 8. Relative abundance of Synergistota linearly decreased with increasing dilution rate (*p* ≤ 0.013), except in incubation 2, transfer 8. The initial inoculum from incubation 1 had a numerically greater relative abundance of Bacteroidota and lower relative abundance of Firmicutes than the inoculum from incubation 2 (Supplementary Figure S18).

High forage incubations were enriched in *Prevotella*, *Streptococcus* (except for incubation 2, transfer 8), Rickenellaceae RC9 group, *Oribacterium* (only incubation 2, transfer 4), and *Succiniclasticum* (except for incubation 2, transfer 8), while high concentrate incubations were enriched in *Escherichia*-*Shigella*, *Pyramidobacter*, *Treponema*, *Selenomonas*, *Succinivibrio*, *Schwartzia*, *Sutterella* (except for incubation 2, transfer 4), and *Anaerovibrio* (except for incubation 1, transfer 8; Supplementary Table S9; Supplementary Figure S19). *Megasphaera* was detected only in incubation 2 and was more abundant in transfer 8 with high concentrate and high dilution rate, with no differences under other conditions or in transfer 4. Prevotellaceae YAB2003, Ga6A1, and UCG-001 (only incubation 2, transfer 4) groups predominated with high concentrate, while Prevotellaceae UCG-003 predominated with the high forage substrate. Of the less represented groups, except for *Enterococcus*, high concentrate incubations had less abundance of *Butyrivibrio*, RF39, Lachnospiraceae UCG-009 and XPB1014, [*Eubacterium*] *ruminantium* and *coprostanoligenes* groups, UCG-002, −009 and −010, *Ruminococcus*, Clostridia vadinBB60 group, MVP-15, NK4A214 group, and *Anaerovorax*.

Dilution rate had a less consistent influence on the overall composition of the bacterial community at the genus level (Supplementary Table S9; Supplementary Figure S19). In incubation 1, transfer 8, high dilution rate cultures with high forage, and low dilution rate with high concentrate each clustered together within their type of substrate. In incubation 2, transfer 4, with the high forage substrate, all three incubations within each dilution rate clustered together, as well as low dilution rate within high concentrate. In incubation 2, transfer 8, high dilution rate incubations within the high concentrate type of substrate clustered together.

The initial inoculum of incubation 1 had numerically greater relative abundance of *Prevotella*, F082, *Fibrobacter*, p-251-o5, Prevotellaceae UCG-001 and UCG-003, [*Eubacterium*] *coprostalonigenes* group, and *Ruminococcus*, and inoculum 2 was higher in *Carnobacterium*, Clostridia UCG-014, Candidatus *Saccharimonas*, *Sutterella*, WCHB1-41, p-251-o5, and RF39 (Supplementary Figure S20). Many genera including *Escherichia coli*-*Shigella*, *Pyramidobacter*, *Streptococcus*, *Schwartzia*, *Selenomonas*, *Sutterella*, *Anaerovibrio*, *Succinivibrio*, *Megasphaera*, and *Oribacterium* that were at less than 0.5% relative abundance in the initial inocula gained importance with one or both substrates throughout the incubations.

### 3.5 Archaeal community composition

A total of 7,700,832 archaeal raw reads were sequenced, with an average of 137,515 reads per sample (137,510 reads for incubation residues and 137,645 for initial inocula), with a minimum of 3,624 and a maximum of 262,616. Total archaeal unique sequence variants were higher with the high forage type of substrate in incubation 1, transfer 8 (*p* = 0.016) and incubation 2, transfer 4, at high dilution rate (*p* < 0.05; interaction substrate by dilution rate *p* = 0.021), but not in incubation 2, transfer 8 (*p* = 0.63; Supplementary Table S7). Archaeal Faith phylogenetic diversity index was greater with high forage in incubation 1, transfer 8 (*p* = 0.005) and at high dilution rate in incubation 2, transfer 4 (*p* < 0.05; interaction substrate by dilution rate *p* = 0.006), and with high concentrate in incubation 2, transfer 8 (*p* = 0.005). Archaeal Shannon diversity index was greater with the high forage substrate in incubation 1, transfer 8 (*p* = 0.004) and incubation 2, transfer 4 (*p* < 0.001), while there were no differences in incubation 2, transfer 8 (*p* = 0.31). Numerically, initial inocula had similar total archaeal unique sequence variants and Shannon diversity indices than their corresponding cultures, and lesser Faith phylogenetic diversity.

The archaeal community composition varied amply and inconsistently between dilution rates, incubations, and transfers (Supplementary Table S10). With the high forage substrate, the relative abundance of *Methanobrevibacter* spp. was low in incubation 1 and numerically higher in incubation 2, although this was numerically and inconsistently affected by dilution rate. With the high concentrate substrate, *Methanobrevibacter* spp. relative abundance was high and numerically increasing with dilution rate in incubation 2, transfer 8, and numerically lower in incubation 1, transfer 8, and in incubation 2, transfer 4. *Methanomicrobium* spp. was also relatively abundant in some incubations and transfers, and numerically decreased with increasing dilution rate. Methanogens from the Methanomassiliicoccales order were generally very abundant, except for the high concentrate substrate at high and mid dilution rates in incubation 2, transfer 8. Methylotrophic methanogens predominated over hydrogenotrophic, except for incubation 2, transfer 8 with high concentrate substrate (Supplementary Figure S21).

Differences between inocula in the archaeal community composition were numerically more pronounced compared to differences in the bacterial community composition. *Methanosphaera* spp., which was virtually absent in the serial incubations, was present in the initial inocula (Supplementary Figure S22). Inoculum 1 had numerically greater relative abundance of *Methanobrevibacter* and Methanomassiliicoccales, while inoculum 2 had numerically greater relative abundance of *Methanomicrobium*, *Methanosphaera*, and unidentified archaea.

## 4 Discussion

Interpretation of the present results should be done within the context that all three dilution rates were high for rumen fermentation; as this study also intended to define a dilution rate for a forthcoming experiment in which the volume transferred was to ideally be minimized, high dilution rates resulted. As a comparison to the dilution rates of 0.14, 0.28, and 0.56 h^−1^ used in the present study, a meta-analysis of the semicontinuous Rusitec fermentation system, and other types of continuous and semicontinuous rumen fermentation systems, reported mean fluid dilution rates of 0.03 ± 0.01 and 0.09 ± 0.05 h^−1^, respectively (Hristov et al., 2012). Numerically lower fluid passage rates than those used in the present study were also reported in a meta-analysis of *in vivo* studies, with mean rumen fluid passage rate in cattle and sheep being 0.052 and 0.050 h^−1^, respectively (Pfau et al., 2023). Furthermore, dilution of the cultures was not constant but occurred every 72 h: in each consecutive 72-h single batch incubation following each successive transfer, there would be curves of microbial growth and production of metabolites until the next transfer, with the most rapidly growing microbes perhaps reaching stationary phase before the next transfer.

### 4.1 Effects of dilution rates on methanogens growth and accumulation of dihydrogen

We hypothesized that, if differences between forages and concentrates in H_2_ accumulation, CH_4_ production, and the acetate to propionate ratio observed *in vivo*, were caused by concentrates having greater dilution rates than forages, those response variables would not differ between rumen serial cultures growing on forages and concentrates at the same dilution rates. The first segment of the model being examined is how dilution rates impacted methanogens growth and H_2_ accumulation. Contrary to our hypothesis, the high concentrate but not the high forage substrate, accumulated substantial H_2_, with the effects of dilution rate being relatively minor. Formate, another electron donor for rumen methanogenesis (Hungate et al., 1970), also accumulated with high concentrate but not with high forage.

The supra-physiological dilution rates used in the present study almost washed-out methanogens with high concentrate but not with the high forage substrate. With high forage, methanogens grew at very rapid rates compared with previous reports of methanogens growing at similar temperature (Supplementary Table S11), were on average nearly four orders of magnitude more abundant than with high concentrate, and their numbers per incubation bottle generally increased throughout the serial incubations (results not shown). One possible explanation for the effect exerted by the type of substrate independently of dilution rate on methanogens growth is that the slightly but consistently lower pH in the high concentrate incubations, decreasing methanogens theoretically maximal growth rate, could have compounded with the high dilution rates (Janssen, 2010), so that methanogens with high concentrate could not grow fast enough to match the high dilution rates used in this study. With some variation among species, rumen methanogens grow optimally between pH 6.0 and 7.5 and some may not grow below pH 6.0 (Smith and Hungate, 1958; Paynter and Hungate, 1968; Jarvis et al., 2000; Rea et al., 2007). However, even at the same pH, accumulation of H_2_ was several-fold higher with high concentrate than with high forage. Working with dual flow rumen fermenters, Wenner et al. (2017) did not find interactions between pH and solids passage rate, and no main effect of solid passage rate, on aqueous H_2_ concentration and gaseous H_2_ and CH_4_ emissions. Furthermore, Wenner et al. (2017) found considerably greater H_2_ emission and aqueous H_2_ concentration at a pH between 6.9 and 6.3, in comparison with a slightly lower pH between 6.4 and 5.8, a result not explained by differences in digestibility or VFA production. Greater H_2_ accumulation at the same pH in our study, and greater H_2_ concentration and emissions at the higher pH in the study of Wenner et al. (2017), do not support the explanation that slightly but consistently lower pH with the high concentrate substrate constrained the theoretically maximal growth rate of methanogens, impeding them to match the high dilution rates utilized.

Greater proportion of CH_4_ produced through the methylotrophic vs. the hydrogenotrophic pathway with the high forage substrate could be another possible explanation for why the incubations with the high forage substrate accumulated little H_2_. Methylotrophic methanogens have a lower thermodynamic threshold for H_2_ than hydrogenotrophic methanogens (Feldewert et al., 2020). This mechanism could perhaps explain greater H_2_ accumulation with high concentrate in incubation 2, transfer 8, in which hydrogenotrophic methanogens predominated with high concentrate, but does not explain the consistently greater accumulation of H_2_ and formate observed with the high concentrate substrate in other transfers and incubations, as the proportion of hydrogenotrophic and methylotrophic methanogens varied widely between substrates and among dilution rates.

Rooke et al. (2014) discussed that rapid fermentation after feeding might temporarily exceed the capacity of methanogens to utilize all the produced H_2_, resulting in the typically observed peaks of H_2_ emission after feed ingestion (Van Lingen et al., 2017). If rapid fermentation conditions are sustained, H_2_ accumulation could further inhibit H_2_ formation and favor the growth of microbial populations incorporating metabolic hydrogen into pathways alternative to methanogenesis (Janssen, 2010). Our experiment was conducted as a series of sequential batch cultures, each of them with its own microbial growth curves. It is possible that the rapid dilution rates used in our study resulted in a close to continuous stage of early digestion and fermentation phases, with rapid H_2_ formation with the high concentrate substrate leading to sustained H_2_ accumulation. Perhaps, most H_2_ and formate accumulation with high concentrate occurred early in the 72 h-incubations and decreased further H_2_ and formate production, as acetate production, a main H_2_- and formate-releasing pathway (as well as the acetate molar ratio; result not shown), was lower with high concentrate than with high forage, and acetate production was negatively related to H_2_ pressure. In contrast, previous results of addition of headspace H_2_ to batch cultures did not find negative effects on acetate molar percentage (Patra and Yu, 2013; Broudiscou et al., 2014; Qiao et al., 2015). Because dissolved H_2_ concentration was not measured in those studies (nor was it in ours), we cannot establish whether the addition of headspace H_2_ reproduces the effect of increased fermentative H_2_ evolution, in terms of dissolved H_2_ concentration.

### 4.2 Effects of accumulation of dihydrogen on methane production and the acetate to propionate ratio

The second segment of the model of fermentation control being examined are the thermodynamic effects of H_2_ and formate accumulation on acetate, H_2_ and CH_4_ production and the acetate to propionate ratio. In our study, accumulation of H_2_ at a partial pressure over ~0.040 atm appeared to limit CH_4_ production, which seems to agree with estimated thermodynamic limits of NADH oxidation to H_2_ in confurcation with reduced ferredoxin (Van Lingen et al., 2016). Accumulation of H_2_ at pressures as high as 0.10 to 0.20 atm with high concentrate in some treatments and transfers (result not shown) does not imply a complete inhibition of H_2_-generating pathways, as H_2_ could still be generated through ferredoxin-based fermentative hydrogenases (Buckel and Thauer, 2013) present in the rumen microbiota (Greening et al., 2019; Pitta et al., 2022). Eventually, lower H_2_ and formate production with high concentrate would have resulted in decreased methanogens growth and CH_4_ production (Janssen, 2010). This alternative explanation inverts the order of the factors in the original model, where, instead of methanogens growth kinetics determining H_2_ accumulation, it would be the fermentative microbiota producing H_2_ at high rates which would result in H_2_ accumulation and thermodynamic inhibition of H_2_ production. This alternative possibility might be less plausible *in vivo*, where passage rates are lower and H_2_ can be released to the atmosphere. This said, dissolved H_2_ has been calculated to be oversaturated in the rumen of sheep even before feeding (Wang et al., 2016), and spatial niches might occur where dissolved H_2_ temporarily inhibits H_2_ production (Janssen, 2010). Comparing fermentation in incubation bottles accumulating and releasing gases at physiological dilution rates could provide insights about the applicability of the present results to *in vivo* situations.

Accumulation of H_2_ and formate did not increase propionate production as an alternative pathway incorporating metabolic hydrogen nor did it decrease the acetate to propionate ratio. We do not regard lactate accumulation to be a consequence of H_2_ accumulation thermodynamically redirecting NADH reoxidation to pyruvate reduction to lactate as an electron sink (Van Lingen et al., 2016; Ungerfeld, 2020), because lactate accumulation was negatively related to H_2_, and with the high forage substrate lactate accumulated with little accumulation of H_2_. Lactate production was instead likely favored by high turnover rates creating a condition of high nutrient availability per unit of time, in which maximizing ATP generation per unit of time was favored over maximizing ATP production per mole of hexose fermented (Russell and Wallace, 1997; see section 4.3). Accumulation of succinate did not seem to be a bottleneck for propionate production, as succinate concentration decreased as the incubations progressed, in agreement with the presence of succinate utilizers *Schwartzia* (van Gylswyk et al., 1997) and *Succiniclasticum* (van Gylswyk, 1995).

The accumulation of H_2_ and formate with high concentrate appeared to enhance the production of butyrate as an alternative electron sink, rather than of propionate. In a previous *in vivo* study, lactate producers *Sharpea* and *Kandleria*, and *Megasphaera*, which metabolizes lactate to butyrate, were abundant in low CH_4_-producing sheep with high rumen turnover rates and high concentration of lactate, in comparison to high CH_4_-producing sheep. A mechanism to explain less CH_4_ production in those sheep based on less H_2_ generation through fermentation of carbohydrates to lactate by *Sharpea* and *Kandleria*, and subsequent lactate metabolism to butyrate by *Megasphaera*, was proposed (Kittelmann et al., 2014; Kamke et al., 2016). In our study, however, *Kandleria* was present at very low numbers (<0.5% of total bacteria), *Sharpea* was not found, and *Megasphaera* was in very low abundance in incubation 1 (results not shown). In addition, most *Megasphaera* strains ferment lactate to greater molar amounts of propionate than of butyrate (Cabral and Weimer, 2023), while increases in propionate paralleling decrease in lactate were not observed in this study. Recently, two bacterial operational taxonomic units with the genetic capacity to produce butyrate, phylogenetically close to *Butyrivibrio fibrisolvens* and *Anaerococcus prevotii*, were identified in rumen cultures growing on lactate (Bandarupalli and St-Pierre, 2023). *Butyrivibrio* spp. were present at moderate abundance in our high forage cultures and could have metabolized some lactate to butyrate but were at very small abundance in our high concentrate incubations, making it unlikely producers of the high amount of butyrate observed with high concentrate. We could not detect the presence of *Anaerococcus* spp. in our cultures. We could not identify any other lactate utilizers known for producing butyrate.

Butyrate could instead have been produced from fermentation of carbohydrates. *Prevotella* produces butyrate as a minor fermentation product (Stewart et al., 1997) and was abundant with the high forage substrate and to a somewhat lesser extent with the high concentrate substrate. Butyrate producer *Megasphaera* was present in incubation 2 but virtually absent in incubation 1, while *Butyrivibrio* was in very small numbers with the high concentrate substrate. Butyrate production has also been reported for some non-ruminal *Treponema* (Lemcke and Burrows, 1981; George and Smibert, 1982), a genus particularly abundant with the high concentrate substrate. At the end, it is unclear which microbial species were responsible for producing important amounts of butyrate, especially with high concentrate, and what caused flows of metabolic hydrogen to be preferentially directed toward butyrate rather than propionate with the high concentrate substrate. Enrichment of butyrate in ^13^C in incubation 2, transfer 8, suggests the possibility of incorporation of CO_2_ into butyrate formation via synthesis of acetyl-CoA (Vital et al., 2014) originated in the Wood-Ljungdahl pathway (Ragsdale and Pierce, 2008). At this point and with this assay performed only in one transfer of serial cultures, that evidence should be considered as highly preliminary and needing confirmation. We did not attempt to conduct an electron balance to quantify the role of butyrate as an electron sink because we did not determine the proportions of hydrogenotrophic and methylotrophic methanogenesis. Methylotrophic methanogenesis was very likely the predominant pathway of CH_4_ production in some treatments, incubations, and transfers in which methylotrophic methanogens of the order Methanomassiliicoccales were dominant.

Previous work with continuous or semicontinuous rumen cultures studying the effect of dilution rate on CH_4_ production and the VFA profile has rendered conflicting results. Isaacson et al. (1975) found that increasing dilution rate decreased CH_4_ and butyrate, and increased propionate per mole of glucose fermented, without altering acetate. Stanier and Davies (1981) also reported a decrease in CH_4_, but an increase in acetate and propionate production with increased dilution rate. Eun et al. (2004) found that increasing concentrate percentage in continuous culture decreased CH_4_ production and the acetate to propionate ratio independently of dilution rate. Martínez et al. (2009) reported that increasing dilution rate did not affect CH_4_ production or acetate molar percentage, increased propionate molar percentage, decreased the acetate to propionate ratio, and decreased butyrate molar percentage with high but not with low retention time of rumen solids. Wenner et al. (2017) found that increasing passage rate in continuous cultures fed a mixed substrate decreased CH_4_ production, the acetate molar percentage, and the acetate to propionate ratio. In the present work, we found a decrease in CH_4_ and acetate production with increasing dilution rate with the high forage substrate only. Lack of effect of dilution rate with high concentrate in our study contrasts with decreased CH_4_ with increasing dilution rate in the study by Isaacson et al. (1975) using glucose, also a rapidly fermentable substrate. Also in contrast with our results, Eun et al. (2004) found increasing CH_4_ production as dilution rate increased from 0.032 to 0.063 h^−1^ with three substrates varying in their forage to concentrate ratio, while CH_4_ production was further increased with the high concentrate substrate only at the highest dilution rate of 0.125 h^−1^. In our study, methanogens were nearly lost with the high concentrate substrate, to the point that changes in dilution rate did not affect CH_4_ production or methanogens numbers.

### 4.3 Bacterial community composition and lactate

The high dilution rates utilized in this study selected for rapidly growing organisms, resulting in an atypical composition of the rumen bacterial community with both types of substrates. In addition, vortexing incubation bottles at the end of each transfer prior to inoculating next transfer is not thought to detach all microbial cells adhered to plant particles, biasing the inoculum toward planktonic and against biofilm microorganisms. In the meta-analysis by Holman and Gzyl (2019), and in the rumen inocula used in incubations 1 and 2, *Escherichia-Shigella* represented less than 0.3% of total bacterial 16S rRNA gene (results not shown). In contrast, after four or eight transfers of serial cultures, *Escherichia*-*Shigella* was the most abundant genus with high concentrate (mean 23.1% of total bacterial 16S rRNA gene), and an important genus with high forage (mean 4.13% of total bacterial 16S rRNA gene). Increased abundance of *Escherichia*-*Shigella* well above its *in vivo* abundance likely obeys to its rapid growth and adaptation to grow in the laboratory (Muñoz-Elías and McKinney, 2006; Orencio-Trejo et al., 2010). *Escherichia coli* does not utilize starch (Ferenci, 1980) and it likely thrived as a rapid, opportunistic cross feeder. When growing anaerobically, *E. coli* ferments sugars to acetate, ethanol, lactate, succinate, H_2_ and formate (Clark, 1989; Pang et al., 2017). The highly abundant *Escherichia*-*Shigella* spp. might have contributed to H_2_ and formate accumulation with high concentrate and lactate accumulation observed with both substrates.

*Pyramidobacter* is also present at low numbers in the rumen (Holman and Gzyl, 2019) and in the initial inocula used in this experiment (results not shown). In contrast, *Pyramidobacter* had a much greater average abundance of 16.3% and 3.51% of total 16S rRNA gene in the high concentrate and high forage serial cultures, respectively. Contrary to the present results, Pan et al. (2017) found a decrease in the abundance of *Pyramidobacter* in the rumen of dairy cows fed a high concentrate diet, which was partially reversed by thiamine supplementation. In a Rusitec study, a *Ginkgo biloba* extract which decreased CH_4_ production and increased H_2_ release, increased *Pyramidobacter* abundance by 41-fold (Oh et al., 2017). If *Pyramidobacter* benefited from H_2_ and formate accumulation, its main electron sinks are unclear, as the only *Pyramidobacter* culture, which was isolated from human oral cavity, produced acetate as its main fermentation product (Downes et al., 2009; Kang et al., 2020). Serial rumen cultures with high transfer rates as used in this study could be used to isolate rumen strains of *Pyramidobacter* for characterizing their physiology.

The observation that *Streptococcus* was more abundant with the high forage than with the high concentrate substrate contrasts with what typically occurs *in vivo*. *Streptococcus bovis* is a rapidly growing bacterium in the rumen of animals fed high concentrate diets (Russell and Robinson, 1984). The rumens of animals fed high forage diets, which are generally associated with relatively slow rumen passage rates (Janssen, 2010) and less nutrient availability, typically harbor lower numbers of *S. bovis* (Russell and Robinson, 1984). *Streptococcus bovis* may have outcompeted slower-growing microorganisms at the high dilution rates used in this study. For example, with a dilution rate as high as 2.040 h^−1^, which is close to four-fold greater the maximum dilution rate used in the present study, *S. bovis* was able to grow at 74% of its maximal growth yield (Russell and Baldwin, 1979). At high dilution rates, *S. bovis* switches from acetate, formate, and ethanol to a homolactic fermentation, even at a pH close to neutrality (Russell and Baldwin, 1979; Silley and Armstrong, 1984). While lactate production generates only two moles of ATP per mole of glucose fermented, compared to four with acetate production, lactate can be produced at a faster rate than acetate, formate, and ethanol, and generate more ATP per unit of time (Russell and Wallace, 1997). As the present study was designed as a succession of serial batch cultures, high dilution rates may contribute to explain lactate accumulation with the high forage substrate, as *Streptococcus* and other lactate producers might have been presented with new substrate before reaching their stationary growth phase. In this regard, decreasing lactate concentration with increasing dilution rate was probably due to the simultaneous decrease in total carbohydrates fermented, as reflected by the decrease in total gas production and total VFA concentration with increasing dilution rate with high forage. *S. bovis* is tolerant to relatively low pH, but its growth rate is maximized at pH 6.0 or higher (Russell et al., 1979; Russell and Dombrowski, 1980), explaining perhaps why it was more abundant with high forage. It should be noted, however, that in a previous study enriching rumen microbial communities using four different types of hemicelluloses as substrates, *Streptococcus* became the dominant genus with nearly 25% relative abundance in cultures enriched with glucomannan from salep tuber (Emerson and Weimer, 2017). Because the enrichments were performed in 48-h batch cultures, *Streptococcus* dominance was not promoted in that study by rapid dilution rates but by the substrate used. Although glucomannans are water-soluble (Razavi et al., 2014) and therefore may differ from the hemicelluloses present in the high forage substrate used in the present study, part of hemicellulose of the high forage substrate digested might have been used by *Streptococcus* through cross-feeding.

*Selenomonas* was abundant in the high concentrate incubations. *Selenomonas* increases its fermentation output of lactate per mole of glucose utilized as growth rate increases (Russell and Baldwin, 1979). *Selenomonas* may have contributed to the observed lactate accumulation, but *Selenomonas* may have also metabolized lactate to acetate and propionate (Russell and Wallace, 1997).

### 4.4 Digestion

The negative DM apparent digestibilities observed in transfer number 8 of both incubations may at first sight be interpreted as small or nil extent of digestion. Negative DM apparent digestibilities seem difficult to reconcile with the relatively high total VFA concentration in transfer number 8 of both incubations, which on average was of 114 and 102 mM for the high forage and high concentrate substrates, respectively (results not shown). In comparison, for example, Hristov et al. (2012) in their meta-analysis reported average VFA concentrations of 78.9 and 93.8 mM for the Rusitec and other rumen continuous and semi-continuous culture systems, respectively, operating at considerably lower dilution rates. The high total VFA concentration along with the negative apparent DM digestibilities can be explained by a preferential partition of fermented organic matter (OM) toward microbial biomass over fermentation products. In most continuous culture studies, increasing dilution rate increased net production of microbial biomass and N, and improved its efficiency of synthesis (Isaacson et al., 1975; Stanier and Davies, 1981; Wenner et al., 2017), because a greater proportion of microbial cells outflows the system before lysing (Wells and Russell, 1996). Although not measured in this study, it seems plausible that the high dilution rates employed herein resulted in relatively high net production of microbial biomass, causing the observed negative apparent digestibilities.

Forages are generally less digested than concentrates in the rumen. In this study, however, disappearance of DM was equal between forages and concentrates in incubation 2, and only slightly less negative with concentrates in incubation 1. Furthermore, total VFA production was greater with the high forage substrate at the low and tended to be greater at the mid dilution rates, with no differences at the high dilution rate. *Streptococcus* and *Prevotella* were abundant and may have had a major role on starch digestion (Avguštin et al., 1997; Stewart et al., 1997). *Prevotella* and *Treponema* may have been involved to different extents in the digestion of hemicellulose (Cotta, 1993; Stewart et al., 1997; Kasperowicz and Míchalowski, 2002; Piknova et al., 2008). *Treponema ruminis*, which has been reported to possess β-glucosidase activity (Newbrook et al., 2017), may have contributed cellulolytic activity if it was present in the incubations. Uncultured Rikenellaceae RC-7 and Bacteroidales UCG-001 predominated with the high forage substrate and have been previously observed to be more abundant in animals fed high forage (Pitta et al., 2010; Bach et al., 2019), and may have also had a role in fiber digestion. Uncultured bacterium F082, Christensenellaceae RC-7, and Lachnospiraceae, which predominated with the high forage substrate in the present study, have on the contrary been found to be associated with an increase in dietary concentrate in yaks (Pang et al., 2022; Yi et al., 2022) and cows (Ricci et al., 2022), thus their possible role in carbohydrates digestion being unclear. Likewise, *Sutterella*, which in our study was more abundant with the high concentrate substrate, was previously reported to be more abundant in grass- than in grain-fed steers (Liu et al., 2022). It is also possible that currently non-described and rapidly growing fibrolytic species were selected by the rapid growing conditions of the cultures and contributed to fiber digestion. Classical cellulolytic bacteria such as *Fibrobacter*, and especially the ruminococci, as well as hemicellulolytic *Butyrivibrio*, were in small numbers. Because of the high rates of cellulose digestion by *F. succinogenes* and the ruminococci (Weimer, 1996), it cannot be discarded that they still played a role in fiber digestion even at the high dilution rates employed in the present study. *Fibrobacter succinogenes* was shown in one study to be able grow on cellobiose at a maximum rate of ~0.27 h^−1^ (Wells and Russell, 1994). It therefore is somewhat surprising that in our study *F. succinogenes* was moderately abundant and that it was not washed out at the high dilution rate of 0.56 h^−1^, particularly considering that it had to digest cellulose forming part of complex plant cell walls. Moreover, selective transfer of fluid containing mostly planktonic organisms and fewer fiber-attached organisms such as classical cellulolytic bacteria (Weimer, 1996), would be thought to further select against *Fibrobacter*. It may be possible that the high transfer rates selected for strains of *Fibrobacter* digesting cellulose at very rapid rates, a possibility that would need to be confirmed conducting isolation studies.

### 4.5 Initial inocula and the incubation effect

For most response variables, the random effect of the incubation interacted with transfer number and type of substrate or dilution rate. In addition, the random effect of the sequence was sometimes significant. This suggests that both variation in the composition of the initial inocula, as well as random inoculation events during the serial incubations, had an influence on the fermentation profile and the composition of the bacterial and archaeal communities. In retrospect, sampling rumen contents before the morning feeding could have decreased the variation between the inocula used in both incubations; on the other hand, it would have likely decreased microbial diversity of the inocula (Yáñez-Ruiz et al., 2016). It seems that differences between the inocula used in both incubations in the relative abundance of bacterial groups did not translate into corresponding differences in the relative abundance of the same groups in transfer 8 of both incubations. For example, numerically higher abundance of *Megasphaera* in incubation 2 compared to incubation 1 was not linked to differences in the initial inocula. Possibly, as rapidly-growing bacteria selected by the rapid dilution rates became dominant, the small initial differences in the abundance of some groups became less apparent. High dilution rates used in this study may have selected for a less diverse bacterial community in comparison with semi-continuous cultures with lower dilution rate or the *in vivo* rumen bacterial community. Less diversity may have in turn resulted in a loss of ecological redundancy (Weimer, 2015), augmenting random variation in fermentation products between incubations and sequences. *In vivo* multiplicity at the levels of rumen microbial taxonomy and metagenomics has been shown to converge into similar metabolic outputs (Taxis et al., 2015), but a very substantial loss of diversity induced by very rapid average dilution rates could perhaps cause larger shifts in the composition of microbial communities and their fermentation products.

It is difficult to understand why the archaeal community composition varied so widely not only between substrates and dilution rates but also between incubations and culture transfers. It is possible that differences between both inocula in the archaeal community composition amplified as incubations progressed and impacted the evolution of utilization and accumulation of H_2_, formate, and methyl groups throughout the incubations, but again, in incubation 2, the archaeal community composition also varied between transfers 4 and 8.

## 5 Conclusion

The accumulation of H_2_ and formate, the production of CH_4_, and the VFA profile of rumen serial mixed cultured was largely determined by the type of substrate independently of dilution rate, disproving our hypothesis. Also, accumulation of H_2_ and formate with the high concentrate substrate was not explained by differences with the high forage substrate in culture pH. It is possible that butyrate production with high concentrate was favored over propionate as an electron sink by bacteria metabolizing lactate to butyrate, although putative bacterial groups which might have conducted that process in both incubations were not identified. Lactate accumulation was favored by the high dilution rates used in this study likely because it allowed for rapid bacterial growth, as the negative relationship between lactate and H_2_ accumulation does not support that lactate accumulated as an electron sink. Factors explaining the wide variation between incubations and transfers in the archaeal community composition remain unclear and require further study.

## Data availability statement

The datasets generated in this study are available at https://www.ncbi.nlm.nih.gov/, under BioProjects PRJNA1063732 and PRJNA1063760 and https://osf.io/5kht9/.

## Ethics statement

The animal study was approved by Comité Institucional de Cuidado Animal, Instituto de Investigaciones Agropecuarias. The study was conducted in accordance with the local legislation and institutional requirements.

## Author contributions

EU: Conceptualization, Data curation, Formal analysis, Funding acquisition, Investigation, Methodology, Project administration, Resources, Supervision, Visualization, Writing – original draft, Writing – review & editing. NC-P: Data curation, Investigation, Writing – review & editing. NV-A: Data curation, Investigation, Writing – review & editing. MSc: Data curation, Investigation, Writing – review & editing. MSa: Data curation, Formal analysis, Investigation, Methodology, Writing – review & editing. LL-P: Investigation, Methodology, Resources, Supervision, Writing – review & editing. MV: Investigation, Methodology, Writing – review & editing. CC: Data curation, Funding acquisition, Investigation, Methodology, Resources, Writing – review & editing. CM: Funding acquisition, Writing – review & editing. NU: Funding acquisition, Writing – review & editing. EM: Data curation, Formal analysis, Funding acquisition, Investigation, Methodology, Resources, Supervision, Writing – review & editing.
