# Supplementary material for: Effects of type of substrate and dilution rate on fermentation in serial rumen mixed cultures
Source: Front Microbiol. 2024 Feb 9;15:1356966. doi: 10.3389/fmicb.2024.1356966 (PMC10883771; doi:10.3389/fmicb.2024.1356966)
Supplement: Supplementary file 1 [file Data_Sheet_1.docx]

Supplementary Material

Effects of type of substrate and dilution rate on fermentation in serial rumen mixed cultures

Emilio M. Ungerfeld*, Nathaly Cancino-Padilla, Nelson Vera-Aguilera, M. Carolina Scorcione, Marcelo Saldivia, Lorena Lagos-Pailla, Milena Vera, Cristián Cerda, Camila Muñoz, Natalie Urrutia, Emilio D. Martínez

Corresponding Author: [emilio.ungerfeld@inia.cl](mailto:emilio.ungerfeld@inia.cl)

**Supplementary Table S1.** Ingredients and proximate composition of the high forage and high concentrate substrates.

| Ingredient (%, DM^1^ basis) | High forage substrate | High concentrate substrate |
| --- | --- | --- |
| Ryegrass hay | 76.7 | 26.5 |
| Corn, ground | 3.8 | 45.0 |
| Oats, ground | 3.8 | 15.1 |
| Soybean meal, solvent-extracted, ground | 15.7 | 13.3 |
| Proximate composition (all % DM basis, except for DM) | | |
| DM (%) | 91.9 | 90.7 |
| OM | 94.2 | 96.4 |
| CP | 14.5 | 14.7 |
| NDF | 48.5 | 24.2 |
| ADF | 29.4 | 12.5 |
| EE | 1.50 | 3.56 |

^1^Abbreviations: ADF = acid detergent fiber; CP = crude protein; DM = dry matter; EE = ether extract; NDF = neutral detergent fiber; OM = organic matter.

**Table S2.** Composition of the growth medium.

| Component | mL/L |
| --- | --- |
| Solution A × 10^1^ | 93.8 |
| Solution B × 10^2^ | 93.8 |
| Distilled water | 753 |
| Trace elements solution^3^ | 0.99 |
| Enriched clarified rumen fluid^4^ | 49.6 |
| 0.1% (m/V) Resazurin solution | 0.99 |
| Reducing solution^5^ | 7.81 |
| 10 N NaOH solution | 0.25 |

^1^Per L distilled water: Na_2_HPO_4_, 8.514 g; KH_2_PO_4_, 13.02 g; MgCl_2_·6H_2_O, 1.05 g (Mould et al., 2005).

^2^Per L distilled water: NaHCO_3_, 54.2 g; NH_4_HCO_3_, 14.1 g (Mould et al., 2005).

^3^Per L distilled water: FeCl_2_.4H_2_O, 1.5 g; CoCl_2_.6H_2_O, 0.19 g; MnCl_2_.4H_2_O, 0.1 g; ZnCl_2_, 0.07 g; H_3_BO_3_, 0.006 g; Na_2_MoO_4_.2H_2_O, 0.036 g; NiCl_2_.6H_2_O, 0.024 g; CuCl_2_.2H_2_O, 0.002 g; 25% (V/V) HCl, 10 mL (Raju, 2016).

^4^Rumen contents were collected before the morning feeding from two ruminally cannulated non-pregnant, non-lactating, Holstein cows. They were strained, and the fluid portion was bubbled with N_2_ for 10 min. It was then centrifuged at 15,500 × g and 4 °C for 30 min under N_2_. The resulting pellet was discarded, and 16.3 g of MgCl_2_.6H_2_O and 11.8 g of CaCl_2_.2H_2_O were added per L of supernatant. Another centrifugation at 15,500 × g and 4 °C for 30 min under N_2_ was conducted and the pellet was discarded. A third and final centrifugation at 15,500 × g and 4 °C for 30 min was conducted. Yeast extract (40 g/L centrifuged rumen fluid) and a filter-sterilized vitamin solution (containing per L distilled water: 4-aminobenzoate, 0.04 g; d-(+)-biotin, 0.010 g; nicotinic acid, 0.1 g; hemicalcium D-(+)-pantothenate, 0.05 g; pyridoxamine hydrochloride, 0.15 g; thiamine chloride hydrochloride, 0.1 g; cyanocobalamin, 0.05 g; D,L-6,8-thioctic acid, 0.03 g; riboflavin, 0.03 g; folic acid, 0.01 g) added at 20 mL/L centrifuged rumen fluid) were added under N_2_. The resulting enriched, clarified rumen fluid was fractioned and stored frozen at -20 °C until used (Raju, 2016).

^5^Containing in total 0.31 g cysteine hydrochloride and 0.31 g Na_2_S.9H_2_O.

**Supplementary Table S3.** Primer sets and PCR conditions used for the quantification of bacterial and archaeal genes.

| Gene | Primer set | Sequence (5’🡪3’) | PCR conditions | Amplicon size (bp) | Reference |
| --- | --- | --- | --- | --- | --- |
| *mrcA* | qmcrA-F | TTCGGTGGATCDCARAGRGC | Initial denaturation step at 95°C for 2 min followed by 40 cycles of 95°C for 15 s, 58ºC for 15s and 72°C for 1 min. | 140 | Denman et al. (2007) |
|  | qmcrA-R | GBARGTCGWAWCCGTAGAATCC |  |  |  |
| 16S rRNA | 1048 F | GTGSTGCAYGGYTGTCGTCA | Initial denaturation step at 95°C for 2 min followed by 40 cycles of 95°C for 15 s, 60ºC for 15s and 72°C for 1 min. | 150 | Maeda et al. (2003) |
|  | 1175 R | ACGTCRTCCMCACCTTCCTC |  |  |  |

**Supplementary Table S4.** Bacterial and archaeal primers used in sequencing 16S rRNA prokaryotic gene.

| Gene | Primer set | Sequence (5’🡪3’) | Reference |
| --- | --- | --- | --- |
| Bacterial 16S rRNA | 515 F | GTGYCAGCMGCCGCGGTAA | Walters et al. (2016) |
|  | 806 R | GGACTACNVGGGTWTCTAAT |  |
| Archaeal 16S rRNA | Arch349 F | CCCTACGGGGTGCASCAG | Mesa et al. (2017) |
|  | Arch806 R | GGACTACVSGGGTATCTAAT |  |

**Supplementary Table S5.** Apparent dry matter disappearance (g/100 g) in serial rumen cultures growing on a high forage or a high concentrate substrate, at three dilution rates.

| Substrate | High forage | | | High concentrate | | | SEM | Substrate *P* = | Dilution rate *P* = | S × D^1^  *P* = |
| --- | --- | --- | --- | --- | --- | --- | --- | --- | --- | --- |
| Dilution rate | Low | Mid | High | Low | Mid | High |  |  |  |  |
| Incubation 1, Transfer 8 | -29.7 | -31.9 | -29.7 | -29.3 | -26.3 | -26.3 | 1.35 | 0.014 | 0.20 | 0.53 |
| Incubation 2, Transfer 4 | 61.1 | 56.7 | 53.2 | 73.7 | 55.9 | 67.0 | 7.29 | 0.18 | 0.33 | 0.58 |
| Incubation 2, Transfer 8 | -4.68 | -3.63 | -5.36 | -3.08 | -3.91 | 1.49 | 2.15 | 0.15 | 0.61 | 0.27 |

^1^S × D = substrate by dilution rate interaction.

**Supplementary Table S6.** Abundance of bacterial 16S rRNA and archaeal *mcrA* gene in serial rumen cultures growing on a high forage or a high concentrate substrate, at three dilution rates.

| Dilution rate | Low | | Mid | | High | | SEM | Substrate *P* = | Dilution rate *P* = | S × D^1^  *P* = |
| --- | --- | --- | --- | --- | --- | --- | --- | --- | --- | --- |
| Substrate | HF^2^ | HC | HF | HC | HF | HC |  |  |  |  |
| Incubation 1, transfer 8 | | | | | | | | | | |
| Bacteria | | | | | | | | | | |
| log 16S rRNA gene copies (g DM^-1^) | 11.1^a3^ | 10.5^b^ | 10.9^p^ | 10.8^p^ | 11.3^x^ | 10.4^y^ | 0.11 | <0.001 | 0.82 | 0.016 |
| log 16S rRNA gene copies (bottle^-1^) | 10.8^a^ | 10.2^b^ | 10.6^p^ | 10.4^p^ | 11.0^x^ | 10.1^y^ | 0.11 | <0.001 | 0.84 | 0.016 |
| Average doubling time beginning of transfer 1 to end of transfer 8 (h) | 6.91^b^ | 7.02^a^ | 3.52^p^ | 3.53^p^ | 1.77^x^ | 1.78^x^ | 0.013 | 0.001 | <0.001 | 0.002 |
| Archaea | | | | | | | | | | |
| log 16S rRNA gene copies (g DM^-1^) | 7.34 | 3.75 | 7.80 | 3.86 | 8.10 | 3.59 | 0.32 | <0.001 | 0.59 | 0.38 |
| log 16S rRNA gene copies (bottle^-1^) | 7.02 | 3.42 | 7.48 | 3.52 | 7.78 | 3.25 | 0.32 | <0.001 | 0.59 | 0.37 |
| Average doubling time beginning of transfer 1 to end of transfer 8 (h) | 7.09^b^ | 7.89^a^ | 3.53^q^ | 3.74^p^ | 1.78^x^ | 1.83^x^ | 0.065 | <0.001 | <0.001 | <0.001 |
| Incubation 2, transfer 4 | | | | | | | | | | |
| Bacteria | | | | | | | | | | |
| log 16S rRNA gene copies (g DM^-1^) | 10.9 | 10.7 | 11.0 | 11.1 | 10.8 | 11.5 | 0.28 | 0.39 | 0.45 | 0.27 |
| log 16S rRNA gene copies (bottle^-1^) | 10.1 | 9.69 | 10.2 | 10.2 | 10.0 | 10.6 | 0.28 | 0.77 | 0.28 | 0.27 |
| Average doubling time beginning of transfer 1 to end of transfer 4 (h) | 6.68 | 6.81 | 3.42 | 3.42 | 1.75 | 1.74 | 0.070 | 0.52 | <0.001 | 0.55 |
| Archaea | | | | | | | | | | |
| log 16S rRNA gene copies (g DM^-1^) | 7.91^a^ | 3.35^b^ | 7.80^p^ | 3.84^q^ | 7.32^x^ | 4.21^y^ | 0.21 | <0.001 | 0.65 | 0.015 |
| log 16S rRNA gene copies (bottle^-1^) | 7.06^a^ | 2.33^b^ | 7.01^p^ | 2.98^q^ | 6.55^x^ | 3.26^y^ | 0.22 | <0.001 | 0.42 | 0.023 |
| Average doubling time beginning of transfer 1 to end of transfer 4 (h) | 6.44^b^ | 8.51^a^ | 3.38^q^ | 3.79^p^ | 1.75^x^ | 1.83^x^ | 0.071 | <0.001 | <0.001 | <0.001 |
| Incubation 2, transfer 8 | | | | | | | | | | |
| Bacteria | | | | | | | | | | |
| log 16S rRNA gene copies (g DM^-1^) | 10.1 | 11.2 | 10.7 | 11.1 | 10.5 | 10.8 | 0.37 | 0.058 | 0.74 | 0.51 |
| log 16S rRNA gene copies (bottle^-1^) | 9.65 | 10.8 | 10.3 | 10.7 | 10.1 | 10.4 | 0.37 | 0.064 | 0.74 | 0.50 |
| Average doubling time beginning of transfer 1 to end of transfer 8 (h) | 7.01^a^ | 6.79^b^ | 3.50^p^ | 3.48^p^ | 1.77^x^ | 1.77^x^ | 0.042 | 0.035 | <0.001 | 0.045 |
| Archaea | | | | | | | | | | |
| log 16S rRNA gene copies (g DM^-1^) | 7.11 | 4.17 | 7.83 | 4.20 | 7.20 | 3.85 | 0.31 | <0.001 | 0.28 | 0.54 |
| log 16S rRNA gene copies (bottle^-1^) | 6.70 | 3.74 | 7.42 | 3.77 | 6.78 | 3.40 | 0.31 | <0.001 | 0.28 | 0.55 |
| Average doubling time beginning of transfer 1 to end of transfer 8 (h) | 6.87^b^ | 7.48^a^ | 3.47^q^ | 3.65^p^ | 1.77^x^ | 1.81^x^ | 0.041 | <0.001 | <0.001 | <0.001 |

^1^S × D = substrate by dilution rate interaction.

^2^HF = high forage; HC = high concentrate.

^3^If there were significant (*P* < 0.05) substrate by dilution rate interactions, the high forage and high concentrate substrates were compared across each dilution rate: a, b = unlike superscripts indicate significant differences within low dilution rate; p, q = unlike superscripts indicate significant differences within medium dilution rate; x, y = unlike superscripts indicate significant differences within high dilution rate.

**Supplementary Table S7.** Diversity indexes of the bacterial and archaeal communities in serial rumen cultures growing on a high forage or a high concentrate substrate, at three dilution rates.

| Dilution rate | Low | | Mid | | High | | SEM | Substrate *P* = | Dilution rate *P* = | S × D^1^  *P* = |
| --- | --- | --- | --- | --- | --- | --- | --- | --- | --- | --- |
| Substrate | HF^2^ | HC | HF | HC | HF | HC |  |  |  |  |
| Incubation 1, transfer 8 | | | | | | | | | | |
| Bacteria | | | | | | | | | | |
| Total unique ASV | 455 | 215 | 447 | 195 | 507 | 182 | 21.3 | <0.001 | 0.56 | 0.14 |
| Faith phylogenetic diversity index | 26.8 | 19.7 | 28.9 | 21.8 | 30.4 | 15.6 | 3.47 | 0.005 | 0.76 | 0.46 |
| Shannon diversity index | 7.59 | 6.33 | 7.45 | 6.58 | 7.83 | 6.44 | 0.095 | <0.001 | 0.22 | 0.041 |
| Archaea | | | | | | | | | | |
| Total unique ASV | 111 | 43 | 99 | 57 | 72 | 53 | 18.6 | 0.016 | 0.65 | 0.44 |
| Faith phylogenetic diversity index | 44.9 | 21.8 | 39.6 | 32.6 | 30.1 | 30.4 | 10.1 | 0.25 | 0.85 | 0.52 |
| Shannon diversity index | 4.67 | 3.41 | 4.87 | 3.61 | 4.10 | 3.58 | 0.35 | 0.004 | 0.55 | 0.49 |
| Incubation 2, transfer 4 | | | | | | | | | | |
| Bacteria | | | | | | | | | | |
| Total unique ASV | 439 | 211 | 396 | 267 | 489 | 228 | 32.5 | <0.001 | 0.57 | 0.15 |
| Faith phylogenetic diversity index | 29.5 | 17.2 | 29.4 | 24.1 | 35.3 | 22.9 | 3.78 | 0.007 | 0.34 | 0.58 |
| Shannon diversity index | 7.55 | 5.98 | 7.56 | 6.64 | 7.80 | 6.08 | 0.16 | <0.001 | 0.14 | 0.057 |
| Archaea | | | | | | | | | | |
| Total unique ASV | 95^a2^ | 59^a^ | 93^p^ | 81^p^ | 157^x^ | 65^y^ | 12.5 | <0.001 | 0.050 | 0.021 |
| Faith phylogenetic diversity index | 28.5^a^ | 34.0^a^ | 25.4^p^ | 43.7^p^ | 61.2^x^ | 33.3^y^ | 6.00 | 0.78 | 0.048 | 0.006 |
| Shannon diversity index | 4.69 | 3.34 | 4.53 | 3.42 | 5.73 | 3.23 | 0.28 | <0.001 | 0.17 | 0.062 |
| Incubation 2, transfer 8 | | | | | | | | | | |
| Bacteria | | | | | | | | | | |
| Total unique ASV | 511 | 322 | 539 | 297 | 496 | 271 | 24.6 | <0.001 | 0.32 | 0.56 |
| Faith phylogenetic diversity index | 31.6 | 20.7 | 30.6 | 22.5 | 33.9 | 19.7 | 2.87 | <0.001 | 0.98 | 0.58 |
| Shannon diversity index | 7.88 | 6.94 | 7.94 | 7.02 | 7.65 | 7.02 | 0.15 | <0.001 | 0.63 | 0.53 |
| Archaea | | | | | | | | | | |
| Total unique ASV | 68 | 66 | 69 | 73 | 81 | 93 | 11.2 | 0.63 | 0.21 | 0.83 |
| Faith phylogenetic diversity index | 10.1 | 26.4 | 17.6 | 33.2 | 22.3 | 50.6 | 7.10 | 0.005 | 0.070 | 0.61 |
| Shannon diversity index | 4.54 | 4.00 | 4.62 | 4.07 | 4.50 | 4.68 | 0.35 | 0.31 | 0.63 | 0.51 |

^1^S × D = substrate by dilution rate interaction.

^2^If there were significant (*P* < 0.05) substrate by dilution rate interactions, the high forage and high concentrate substrates were compared across each dilution rate: a, b = unlike superscripts indicate significant differences within low dilution rate; p, q = unlike superscripts indicate significant differences within medium dilution rate; x, y = unlike superscripts indicate significant differences within high dilution rate.

**Supplementary Table S8.** Relative abundance (% of total 16S rRNA gene) of bacterial phyla in serial rumen cultures growing on a high forage or a high concentrate substrate, at three dilution rates. Phyla are listed in alphabetical order^1^

| Dilution rate | Low | | Mid | | High | | SEM | Substrate *P* = | Dilution rate *P* = | S × D  *P* = |
| --- | --- | --- | --- | --- | --- | --- | --- | --- | --- | --- |
| Substrate | HF^2^ | HC | HF | HC | HF | HC |  |  |  |  |
| Incubation 1, transfer 8 | | | | | | | | | | |
| Actinobacteriota | 1.05 | 0.19 | 0.94 | 0.074 | 1.71 | 0.017 | 0.22 | < 0.001 | 0.28 | 0.13 |
| Bacteroidota | 41.7 | 18.3 | 35.1 | 29.3 | 31.5 | 27.5 | 4.45 | 0.010 | 0.81 | 0.094 |
| Desulfobacterota | 0.64 | 0.74 | 0.40 | 1.18 | 0.61 | 1.01 | 0.20 | 0.024 | 0.82 | 0.28 |
| Fibrobacterota | 0.36^b4^ | 0.84^a^ | 0.41^p^ | 0.019^p^ | 0.45^x^ | 0.092^x^ | 0.15 | 0.47 | 0.048 | 0.018 |
| Firmicutes | 35.6 | 17.1 | 47.9 | 22.7 | 48.3 | 14.0 | 4.66 | < 0.001 | 0.20 | 0.27 |
| Fusobacteriota | 0.67 | 1.04 | 0.58 | 1.10 | 0.65 | 1.18 | 0.38 | 0.15 | 0.98 | 0.97 |
| Proteobacteria | 6.95 | 27.3 | 3.82 | 23.4 | 5.33 | 32.5 | 2.87 | < 0.001 | 0.21 | 0.38 |
| Spirochaetota | 4.71 | 6.00 | 5.05 | 5.13 | 3.12 | 8.02 | 1.45 | 0.10 | 0.95 | 0.27 |
| Synergistota | 4.31 | 27.5 | 2.48 | 15.6 | 3.61 | 14.6 | 2.54 | < 0.001 | 0.029^3^ | 0.073 |
| Verrucomicrobiota | 2.84 | 0.54 | 2.32 | 0.48 | 3.15 | 0.60 | 0.31 | < 0.001 | 0.35 | 0.53 |
| Incubation 2, transfer 4 | | | | | | | | | | |
| Actinobacteriota | 2.17 | 0.18 | 2.61 | 0.35 | 2.54 | 0.29 | 0.27 | < 0.001 | 0.50 | 0.85 |
| Bacteroidota | 41.9 | 13.8 | 36.6 | 18.3 | 35.0 | 14.1 | 3.56 | < 0.001 | 0.62 | 0.40 |
| Desulfobacterota | 0.52 | 0.70 | 0.30 | 0.69 | 0.22 | 0.72 | 0.14 | 0.008 | 0.58 | 0.53 |
| Fibrobacterota | 0.32 | 0.34 | 0.44 | 1.25 | 0.41 | 0.87 | 0.20 | 0.022 | 0.075 | 0.19 |
| Firmicutes | 36.6 | 16.1 | 42.4 | 24.4 | 42.9 | 19.7 | 3.56 | < 0.001 | 0.17 | 0.77 |
| Fusobacteriota | ND^5^ | 0.011 | 0.020 | ND | ND | ND | 0.010 | 0.71 | 0.58 | 0.28 |
| Proteobacteria | 6.53 | 40.4 | 6.14 | 32.8 | 8.05 | 45.7 | 3.84 | < 0.001 | 0.20 | 0.38 |
| Spirochaetota | 2.56 | 3.36 | 3.52 | 6.17 | 2.94 | 4.11 | 0.71 | 0.021 | 0.055 | 0.41 |
| Synergistota | 4.78^b^ | 24.6^a^ | 4.26^q^ | 15.2^p^ | 5.00^y^ | 14.1^x^ | 1.59 | < 0.001 | 0.011 | 0.012 |
| Verrucomicrobiota | 2.59 | 0.21 | 2.04 | 0.66 | 1.94 | 0.31 | 0.23 | < 0.001 | 0.47 | 0.12 |
| Incubation 2, transfer 8 | | | | | | | | | | |
| Actinobacteriota | 3.42 | 1.03 | 2.00 | 1.33 | 1.39 | 1.66 | 0.69 | 0.13 | 0.58 | 0.19 |
| Bacteroidota | 37.9 | 23.8 | 38.9 | 20.3 | 40.8 | 17.6 | 3.02 | < 0.001 | 0.85 | 0.36 |
| Desulfobacterota | 0.67 | 0.73 | 0.59 | 0.60 | 0.33 | 0.44 | 0.082 | 0.39 | 0.007^3^ | 0.80 |
| Fibrobacterota | 0.70 | 1.43 | 0.98 | 1.52 | 0.83 | 1.89 | 0.48 | 0.071 | 0.83 | 0.87 |
| Firmicutes | 37.1 | 47.9 | 39.0 | 43.8 | 42.6 | 31.5 | 6.34 | 0.77 | 0.67 | 0.24 |
| Fusobacteriota | ND | 0.021 | ND | ND | ND | ND | 0.008 | 0.34 | 0.40 | 0.40 |
| Proteobacteria | 6.33^a^ | 5.81^a^ | 5.88^p^ | 13.1^p^ | 2.26^y^ | 25.3^x^ | 2.89 | 0.001 | 0.061 | 0.005 |
| Spirochaetota | 3.59 | 6.52 | 4.26 | 5.93 | 5.48 | 7.07 | 1.29 | 0.074 | 0.58 | 0.85 |
| Synergistota | 5.43 | 11.0 | 2.76 | 11.6 | 2.20 | 13.8 | 2.90 | 0.003 | 0.94 | 0.60 |
| Verrucomicrobiota | 2.84 | 1.51 | 3.56 | 1.57 | 2.90 | 0.58 | 0.57 | 0.002 | 0.37 | 0.68 |

^1^Only phyla with a relative abundance of 0.5% of total bacterial 16S rRNA gene or greater in at least one level of a main effect in at least one incubation and transfer are presented.

^2^HF = high forage; HC = high concentrate; S × D = substrate by dilution rate interaction.

^3^Linear response to dilution rate.

^4^If there were significant (*P* < 0.05) substrate by dilution rate interactions, substrates were compared across each dilution rate: a, b = unlike superscripts indicate significant differences within low dilution rate; p, q = unlike superscripts indicate significant differences within medium dilution rate; x, y = unlike superscripts indicate significant differences within high dilution rate.

^5^ND = not detected.

**Supplementary Table S9.** Relative abundance (% of total 16S rRNA gene) of bacterial genera in serial rumen cultures growing on a high forage or a high concentrate substrate, at three dilution rates. Genera are listed in alphabetical order^1^.

| Dilution rate | Low | | Mid | | High | | SEM | Substrate *P* = | Dilution rate *P* = | S × D  *P* = |
| --- | --- | --- | --- | --- | --- | --- | --- | --- | --- | --- |
| Substrate | HF^2^ | HC | HF | HC | HF | HC |  |  |  |  |
| Incubation 1, transfer 8 | | | | | | | | | | |
| *Anaerobiospirillum* | 0.31 | 0.81 | 0.22 | 1.31 | 0.18 | 2.17 | 0.51 | 0.015 | 0.50 | 0.38 |
| *Anaeroplasma* | 1.48 | 1.33 | 1.49 | 2.23 | 1.51 | 3.61 | 1.06 | 0.32 | 0.56 | 0.58 |
| *Anaerovibrio* | 1.90 | 2.57 | 3.56 | 3.51 | 3.38 | 2.84 | 0.37 | 0.93 | 0.013^3^ | 0.29 |
| *Anaerovorax* | 0.17^a^ | 0.0095^b^ | 0.12^p^ | ND^q^ | 0.61^x^ | ND^y^ | 0.035 | <0.001 | <0.001 | <0.001 |
| *Asteroleplasma* | 0.087 | 0.36 | 0.15 | 1.04 | 0.31 | 0.53 | 0.24 | 0.033 | 0.32 | 0.32 |
| Bacteroidales UCG-001 | 2.55 | 0.30 | 2.17 | 0.20 | 1.83 | 0.079 | 0.59 | 0.001 | 0.73 | 0.91 |
| *Butyrivibrio* | 0.32 | 0.0028 | 0.11 | 0.0044 | 0.67 | ND | 0.092 | <0.001 | 0.032 | 0.030 |
| Christensenellaceae R-7 group | 0.71 | 0.015 | 0.58 | 0.12 | 0.65 | 0.099 | 0.13 | <0.001 | 0.98 | 0.65 |
| Clostridia vadinBB60 group | 1.52 | 0.21 | 1.77 | 0.39 | 1.85 | ND | 0.23 | <0.001 | 0.96 | 0.48 |
| *Clostridium* senso stricto 1 | 0.054 | 1.00 | 0.040 | 1.07 | 0.029 | 0.96 | 0.20 | <0.001 | 0.95 | 0.96 |
| *Desulfovibrio* | 0.57 | 0.74 | 0.37 | 1.18 | 0.58 | 1.01 | 0.20 | 0.014 | 0.78 | 0.31 |
| *Enterococcus* | 0.040^b^ | 0.83^a^ | 0.036^q^ | 1.16^p^ | 0.014^y^ | 0.56^x^ | 0.080 | <0.001 | 0.0084 | 0.013 |
| *Escherichia-Shigella* | 5.36 | 21.0 | 2.27 | 13.3 | 3.89 | 24.7 | 3.26 | <0.001 | 0.15 | 0.40 |
| [*Eubacterium*] *coprostanoligenes* group | 0.39 | ND | 0.075 | ND | 0.56 | 0.0076 | 0.11 | 0.002 | 0.098 | 0.11 |
| F082 | 1.88 | 0.16 | 1.38 | 0.0076 | 1.03 | ND^5^ | 0.26 | <0.001 | 0.18 | 0.43 |
| *Fibrobacter* | 0.36^b^ | 0.84^a^ | 0.41^p^ | 0.019^p^ | 0.46^x^ | 0.092^x^ | 0.15 | 0.46 | 0.048 | 0.018 |
| *Fusobacterium* | 0.67 | 1.04 | 0.58 | 1.10 | 0.65 | 1.18 | 0.38 | 0.15 | 0.98 | 0.97 |
| *Lachnoclostridium* | 0.59 | 0.29 | 0.72 | 0.53 | 0.46 | 0.14 | 0.12 | 0.019 | 0.060 | 0.87 |
| Lachnospiraceae UCG-009 | 0.38 | ND | 1.90 | ND | 0.49 | ND | 0.45 | 0.027 | 0.20 | 0.20 |
| *Olsenella* | 0.33 | 0.18 | 0.33 | 0.060 | 0.82 | ND | 0.14 | 0.004 | 0.32 | 0.076 |
| *Oribacterium* | 1.70 | 0.33 | 3.08 | 5.36 | 0.80 | 0.27 | 1.30 | 0.91 | 0.031 | 0.37 |
| p-251-o5 | 0.77 | 0.089 | 0.79 | 0.041 | 1.07 | 0.018 | 0.098 | <0.001 | 0.38 | 0.17 |
| *Parabacteroides* | 0.16 | ND | 0.36 | 1.22 | 0.12 | 0.40 | 0.28 | 0.17 | 0.061 | 0.21 |
| *Paraprevotella* | 0.18 | 1.07 | 0.39 | 1.50 | 0.51 | 2.27 | 0.52 | 0.012 | 0.36 | 0.70 |
| *Prevotella* | 18.2^a4^ | 9.04^b^ | 13.6^p^ | 7.84^p^ | 3.88^x^ | 9.47^x^ | 2.78 | 0.19 | 0.079 | 0.050 |
| Prevotellaceae Ga6A1 group | 0.13 | 1.14 | 0.087 | 0.70 | 0.28 | 2.11 | 0.74 | 0.081 | 0.55 | 0.71 |
| Prevotellaceae UCG-001 | 0.52 | 0.52 | 0.26 | 0.12 | 017 | 0.39 | 0.11 | 0.82 | 0.031^3^ | 0.31 |
| Prevotellaceae UCG-003 | 0.95 | 0.39 | 0.90 | 0.20 | 1.27 | 0.35 | 0.15 | <0.001 | 0.27 | 0.50 |
| Prevotellaceae YAB2003 group | 0.33 | 1.66 | 0.47 | 4.82 | 0.54 | 4.47 | 0.75 | <0.001 | 0.088 | 0.13 |
| *Pyramidobacter* | 3.92 | 27.4 | 2.23 | 15.5 | 3.21 | 14.6 | 2.55 | <0.001 | 0.032^3^ | 0.076 |
| RF39 | 0.39 | 0.0027 | 0.14 | 0.016 | 0.51 | 0.0032 | 0.11 | 0.002 | 0.29 | 0.24 |
| Rikenellaceae RC9 gut group | 15.0 | 3.34 | 13.6 | 11.2 | 19.5 | 6.24 | 3.35 | 0.006 | 0.50 | 0.26 |
| *Schwartzia* | 0.73^b^ | 3.39^a^ | 0.72^q^ | 2.92^p^ | 1.41^x^ | 1.78^x^ | 0.33 | <0.001 | 0.39 | 0.010 |
| *Selenomonas* | 0.53 | 1.58 | 0.21 | 2.44 | 0.021 | 1.66 | 0.30 | <0.001 | 0.31 | 0.19 |
| *Sphaerochaeta* | 0.84 | 0.48 | 1.24 | 0.78 | 0.94 | 0.66 | 0.31 | 0.18 | 0.55 | 0.96 |
| *Streptococcus* | 10.5 | 0.97 | 21.6 | 0.50 | 20.1 | 0.34 | 4.37 | <0.001 | 0.45 | 0.38 |
| *Succiniclasticum* | 0.79 | ND | 0.35 | 0.071 | 0.82 | 0.0084 | 0.13 | <0.001 | 0.27 | 0.11 |
| *Succinivibrio* | 0.34^a^ | 2.42^a^ | 0.12^q^ | 5.72^p^ | 0.15^x^ | 1.86^x^ | 0.69 | <0.001 | 0.037 | 0.028 |
| *Sutterella* | 0.44 | 3.04 | 1.11 | 3.00 | 0.96 | 3.72 | 0.32 | <0.001 | 0.21 | 0.39 |
| *Treponema* | 3.87 | 5.52 | 3.82 | 4.35 | 2.18 | 7.35 | 1.37 | 0.049 | 0.86 | 0.25 |
| UCG-002 | 0.53 | 0.0097 | 0.42 | ND | 0.32 | 0.0067 | 0.076 | <0.001 | 0.38 | 0.41 |
| UCG-004 | 3.24 | 2.02 | 2.62 | 0.38 | 2.02 | 0.59 | 0.43 | <0.001 | 0.021^3^ | 0.49 |
| UCG-009 | 0.67 | 0.0042 | 0.083 | 0.033 | 0.17 | ND | 0.15 | 0.032 | 0.16 | 0.13 |
| UCG-010 | 0.82^a^ | 0.014^b^ | 0.52^p^ | 0.0044^q^ | 1.73^x^ | 0.0028^y^ | 0.16 | <0.001 | 0.006 | 0.006 |
| WCHB1-41 | 2.27 | 0.53 | 1.97 | 0.48 | 2.88 | 0.60 | 0.32 | <0.001 | 0.29 | 0.46 |
| Incubation 2, transfer 4 | | | | | | | | | | |
| *Anaerobiospirillum* | 0.37 | 0.49 | 0.56 | 0.60 | 0.41 | 0.51 | 0.078 | 0.21 | 0.16 | 0.90 |
| *Anaeroplasma* | 1.65 | 0.63 | 1.15 | 0.60 | 1.16 | 0.61 | 0.20 | <0.001 | 0.37 | 0.42 |
| *Anaerovibrio* | 0.36 | 2.17 | 0.40 | 3.45 | 0.70 | 3.30 | 0.31 | <0.001 | 0.071 | 0.18 |
| *Anaerovorax* | 0.60^a^ | 0.0053^b^ | 0.45^p^ | 0.0017^q^ | 0.30^x^ | 0.0053^y^ | 0.043 | <0.001 | 0.014 | 0.014 |
| *Enterococcus* | 0.025 | 0.58 | 0.018 | 0.26 | 0.040 | 0.52 | 0.095 | <0.001 | 0.21 | 0.26 |
| Bacteroidales UCG-001 | 1.09 | 0.38 | 1.14 | 0.37 | 1.33 | 0.29 | 0.19 | <0.001 | 0.91 | 0.66 |
| *Butyrivibrio* | 0.21 | ND | 0.69 | 0.014 | 0.66 | 0.0054 | 0.21 | 0.012 | 0.45 | 0.48 |
| Clostridia vadinBB60 group | 1.89 | 0.019 | 1.71 | 0.026 | 1.26 | 0.021 | 0.14 | <0.001 | 0.096 | 0.097 |
| *Clostridium* senso stricto 1 | 0.37 | 0.61 | 0.25 | 0.67 | 0.18 | 0.34 | 0.11 | 0.012 | 0.13 | 0.51 |
| *Denitrobacterium* | 0.070 | ND | 0.35 | 0.11 | 0.56 | 0.016 | 0.073 | <0.001 | 0.012 | 0.022 |
| *Desulfovibrio* | 0.50 | 0.70 | 0.25 | 0.69 | 0.18 | 0.72 | 0.14 | 0.004 | 0.53 | 0.48 |
| *Escherichia-Shigella* | 4.79 | 38.9 | 4.37 | 30.8 | 6.06 | 43.0 | 4.20 | <0.001 | 0.29 | 0.46 |
| [*Eubacterium*] *coprostalinogenes* | 0.92^a^ | 0.0080^b^ | 0.67^p^ | ND^q^ | 0.055^x^ | ND^x^ | 0.15 | <0.001 | 0.034 | 0.037 |
| F082 | 0.86 | ND | 1.51 | 0.029 | 2.22 | 0.065 | 0.23 | <0.001 | 0.026 | 0.043 |
| *Fibrobacter* | 0.31 | 0.34 | 0.44 | 1.25 | 0.41 | 0.87 | 0.20 | 0.022 | 0.071 | 0.20 |
| Lachnoclostridium | 0.59 | 0.86 | 0.76 | 1.43 | 1.26 | 1.46 | 0.20 | 0.038 | 0.023^3^ | 0.48 |
| Lachnospiraceae NK4A136 | 1.55 | 0.054 | 0.95 | 0.11 | 2.43 | ND | 0.75 | 0.024 | 0.67 | 0.58 |
| *Megasphaera* | 0.52 | 0.68 | 0.58 | 1.20 | 1.04 | 1.07 | 0.26 | 0.23 | 0.24 | 0.51 |
| *Olsenella* | 0.97 | 0.15 | 1.19 | 0.23 | 1.12 | 0.21 | 0.19 | <0.001 | 0.72 | 0.93 |
| *Oribacterium* | 1.20 | 0.51 | 1.46 | 1.07 | 1.89 | 0.63 | 0.23 | 0.001 | 0.17 | 0.19 |
| Lachnospiraceae XPB1014 group | 0.50 | 0.019 | 0.72 | ND | 0.26 | 0.0018 | 0.15 | 0.002 | 0.37 | 0.36 |
| NK4A214 group | 0.72^a^ | ND^b^ | 0.38^p^ | ND^q^ | 0.32^x^ | 0.0098^y^ | 0.065 | <0.001 | 0.019 | 0.016 |
| p-251-o5 | 0.68 | 0.41 | 0.91 | 0.28 | 1.19 | 0.28 | 0.16 | <0.001 | 0.50 | 0.17 |
| *Prevotella* | 13.1 | 3.98 | 14.4 | 4.99 | 10.0 | 3.92 | 1.79 | <0.001 | 0.34 | 0.61 |
| Prevotellaceae Ga6A1 group | 0.0017 | 0.59 | 0.18 | 0.55 | 0.065 | 0.38 | 0.071 | <0.001 | 0.17 | 0.17 |
| Prevotellaceae UCG-001 | 0.19 | 0.72 | 0.21 | 0.90 | 0.26 | 0.48 | 0.10 | <0.001 | 0.24 | 0.098 |
| Prevotellaceae UCG-003 | 0.85 | ND | 0.62 | 0.062 | 0.66 | 0.024 | 0.062 | <0.001 | 0.32 | 0.085 |
| Prevotellaceae YAB2003 | 0.17 | 1.38 | 0.26 | 1.36 | 0.25 | 0.89 | 0.22 | <0.001 | 0.52 | 0.42 |
| *Pyramidobacter* | 4.54 | 24.5 | 3.74 | 15.0 | 4.57 | 14.1 | 1.59 | <0.001 | 0.010^3^ | 0.015 |
| RF39 | 1.14 | 0.054 | 1.16 | 0.025 | 0.87 | 0.013 | 0.20 | <0.001 | 0.67 | 0.75 |
| Rikenellaceae RC9 group | 23.9^a^ | 4.87^b^ | 15.7^p^ | 7.90^q^ | 17.5^x^ | 6.46^y^ | 1.61 | <0.001 | 0.24 | 0.013 |
| *Schwartzia* | 0.78 | 3.07 | 0.68 | 3.11 | 1.78 | 2.94 | 0.40 | <0.001 | 0.45 | 0.26 |
| *Selenomonas* | 0.13^a^ | 2.17^a^ | 0.20^q^ | 6.78^p^ | 0.20^y^ | 5.50^x^ | 0.68 | <0.001 | 0.013 | 0.016 |
| *Sphaerochaeta* | 0.99 | 0.017 | 0.90 | ND | 0.54 | 0.056 | 0.039 | <0.001 | <0.001 | <0.001 |
| *Streptococcus* | 10.4 | 1.00 | 14.7 | 0.48 | 12.3 | 0.41 | 3.67 | 0.002 | 0.87 | 0.81 |
| *Succiniclasticum* | 0.93 | 0.38 | 1.29 | 0.45 | 1.57 | 0.34 | 0.21 | <0.001 | 0.38 | 0.32 |
| *Succinivibrio* | 0.13 | 0.70 | 0.26 | 0.96 | 0.41 | 1.75 | 0.37 | 0.015 | 0.23 | 0.56 |
| *Sutterella* | 0.31 | 0.23 | 0.27 | 0.39 | 0.61 | 0.42 | 0.073 | 0.43 | 0.013^3^ | 0.14 |
| *Treponema* | 1.32 | 3.33 | 2.63 | 6.12 | 2.35 | 3.99 | 0.71 | 0.001 | 0.040 | 0.41 |
| UCG-004 | 1.04 | 0.57 | 0.82 | 0.86 | 1.01 | 0.60 | 0.20 | 0.11 | 0.97 | 0.40 |
| UCG-010 | 1.18^a^ | 0.021^b^ | 0.94^p^ | 0.0066^q^ | 0.50^x^ | ND^y^ | 0.11 | <0.001 | 0.023 | 0.032 |
| WCHB1-41 | 2.14 | 0.21 | 1.78 | 0.66 | 1.74 | 0.31 | 0.23 | <0.001 | 0.68 | 0.25 |
| Incubation 2, transfer 8 | | | | | | | | | | |
| *Anaeroplasma* | 1.17^a^ | 1.28^a^ | 1.94^p^ | 1.24^q^ | 0.66^y^ | 1.56^x^ | 0.22 | 0.58 | 0.13 | 0.013 |
| *Anaerovibrio* | 0.35 | 3.17 | 0.34 | 3.19 | 0.57 | 3.19 | 0.47 | <0.001 | 0.96 | 0.96 |
| *Anaerovorax* | 0.57 | 0.0081 | 0.59 | ND | 0.64 | ND | 0.040 | <0.001 | 0.76 | 0.66 |
| *Asteroleplasma* | 0.21 | 0.44 | 0.26 | 0.38 | 0.19 | 0.54 | 0.17 | 0.11 | 0.97 | 0.80 |
| Bacteroidales UCG-001 | 1.33 | 0.22 | 1.13 | 0.25 | 1.27 | 0.27 | 0.21 | <0.001 | 0.91 | 0.87 |
| *Bacteroides* | 0.050 | 0.33 | 0.19 | 1.33 | 0.024 | 0.92 | 0.18 | <0.001 | 0.029 | 0.095 |
| *Butyrivibrio* | 0.65 | 0.15 | 0.42 | 0.18 | 1.01 | 0.0052 | 0.22 | 0.007 | 0.64 | 0.25 |
| Christensenellaceae R-7 group | 1.62 | 0.062 | 0.69 | 0.13 | 1.13 | 0.0033 | 0.32 | 0.001 | 0.41 | 0.31 |
| *Clostridia* vadinBB60 group | 0.99 | 0.036 | 2.20 | 0.16 | 1.41 | 0.26 | 0.23 | <0.001 | 0.042 | 0.080 |
| *Denitrobacterium* | 0.21 | 0.15 | 0.19 | 0.38 | 0.24 | 0.52 | 0.081 | 0.061 | 0.10 | 0.14 |
| *Desulfovibrio* | 0.59 | 0.68 | 0.55 | 0.60 | 0.31 | 0.44 | 0.086 | 0.21 | 0.028^3^ | 0.89 |
| *Escherichia-Shigella* | 4.92^a^ | 4.03^a^ | 4.60^p^ | 9.34^p^ | 0.88^y^ | 23.1^x^ | 2.77 | 0.002 | 0.051 | 0.003 |
| [*Eubacterium*] *coprostanoligenes* group | 0.73 | 0.038 | 0.85 | 0.057 | 0.58 | 0.0039 | 0.19 | <0.001 | 0.70 | 0.85 |
| [*Eubacterium*] *ruminantium* group | 0.032 | ND | 0.082 | 0.039 | 1.05 | ND | 0.30 | 0.15 | 0.21 | 0.19 |
| F082 | 1.17 | 0.070 | 1.98 | 0.062 | 1.16 | 0.14 | 0.22 | <0.001 | 0.17 | 0.12 |
| *Fibrobacter* | 0.68 | 1.43 | 0.93 | 1.52 | 0.84 | 1.89 | 0.48 | 0.065 | 0.82 | 0.89 |
| *Lachnoclostridium* | 1.04 | 1.15 | 0.72 | 1.08 | 1.04 | 1.10 | 0.16 | 0.21 | 0.45 | 0.62 |
| Lachnospiraceae NK4A136 | 1.07 | 0.068 | 1.33 | 0.066 | 1.54 | 0.048 | 0.41 | 0.003 | 0.86 | 0.84 |
| *Megasphaera* | 1.87^a^ | 1.25^a^ | 2.36^p^ | 2.37^p^ | 1.32^y^ | 3.21^x^ | 0.44 | 0.26 | 0.17 | 0.036 |
| MVP-15 | 0.17 | ND | 0.59 | 0.0064 | 0.31 | ND | 0.13 | 0.005 | 0.28 | 0.30 |
| NK4A214 group | 0.70 | 0.0073 | 0.49 | ND | 0.43 | 0.010 | 0.080 | <0.001 | 0.24 | 0.24 |
| *Olsenella* | 2.06 | 0.55 | 0.91 | 0.77 | 0.46 | 0.91 | 0.52 | 0.37 | 0.48 | 0.19 |
| *Oribacterium* | 1.97 | 1.90 | 1.00 | 1.23 | 1.14 | 1.22 | 0.39 | 0.81 | 0.11 | 0.93 |
| *Prevotella* | 18.2 | 11.4 | 17.3 | 8.60 | 22.8 | 7.22 | 2.09 | <0.001 | 0.58 | 0.13 |
| Prevotellaceae Ga6A1 group | 0.056 | 0.65 | 0.16 | 0.58 | 0.29 | 0.58 | 0.12 | <0.001 | 0.75 | 0.48 |
| Prevotellaceae UCG-001 | 0.35 | 0.76 | 0.17 | 0.47 | 0.35 | 0.32 | 0.25 | 0.29 | 0.58 | 0.65 |
| Prevotellaceae UCG-003 | 0.62^a^ | 0.28^a^ | 0.95^p^ | 0.26^q^ | 1.28^x^ | 0.21^y^ | 0.11 | <0.001 | 0.063 | 0.023 |
| Prevotellaceae YAB2003 group | 0.19 | 1.13 | 0.14 | 0.54 | 0.21 | 1.44 | 0.29 | 0.003 | 0.27 | 0.36 |
| *Pyramidobacter* | 5.00 | 10.8 | 2.53 | 11.4 | 1.83 | 13.7 | 2.87 | 0.003 | 0.94 | 0.59 |
| RF39 | 1.25 | 0.030 | 0.70 | 0.023 | 0.99 | 0.026 | 0.22 | <0.001 | 0.48 | 0.50 |
| Rikenellaceae RC9 group | 13.7 | 6.30 | 14.8 | 5.70 | 11.3 | 4.35 | 1.68 | <0.001 | 0.32 | 0.81 |
| *Ruminococcus* | 0.13 | ND | 0.28 | ND | 0.54 | ND | 0.11 | 0.005 | 0.24 | 0.24 |
| *Schwartzia* | 1.01 | 1.09 | 1.21 | 3.09 | 0.55 | 3.99 | 0.68 | 0.007 | 0.19 | 0.085 |
| *Selenomonas* | 0.32 | 1.04 | 0.23 | 1.74 | 0.14 | 2.38 | 0.57 | 0.008 | 0.60 | 0.43 |
| *Sphaerochaeta* | 0.89 | 0.10 | 1.27 | 0.036 | 0.85 | 0.24 | 0.14 | <0.001 | 0.54 | 0.11 |
| *Streptococcus* | 7.22 | 26.9 | 9.89 | 21.1 | 14.4 | 6.40 | 7.24 | 0.22 | 0.64 | 0.19 |
| *Succiniclasticum* | 1.49 | 1.58 | 0.84 | 1.67 | 0.98 | 1.28 | 0.34 | 0.16 | 0.49 | 0.55 |
| *Succinivibrio* | 0.085 | 0.70 | 0.038 | 1.12 | 0.046 | 0.85 | 0.32 | 0.008 | 0.84 | 0.78 |
| *Sutterella* | 0.28^a^ | 0.18^a^ | 0.26^q^ | 1.62^p^ | 0.53^x^ | 0.60^x^ | 0.17 | 0.007 | 0.004 | 0.002 |
| *Treponema* | 2.54 | 6.42 | 2.41 | 5.89 | 4.34 | 6.82 | 1.25 | 0.007 | 0.51 | 0.85 |
| UCG-002 | 0.69 | 0.11 | 0.51 | 0.11 | 0.70 | 0.0065 | 0.15 | <0.001 | 0.82 | 0.61 |
| UCG-004 | 1.26 | 3.16 | 1.63 | 1.81 | 0.95 | 2.11 | 0.42 | 0.009 | 0.29 | 0.17 |
| UCG-010 | 1.02 | 0.018 | 1.28 | 0.0072 | 0.94 | 0.0091 | 0.12 | <0.001 | 0.38 | 0.36 |
| WCHB1-41 | 2.52 | 1.51 | 3.14 | 1.57 | 2.75 | 0.57 | 0.56 | 0.005 | 0.48 | 0.59 |

^1^Only phyla with a relative abundance of 0.5% of total bacterial 16S rRNA gene or greater in at least one level of a main effect in at least one incubation and transfer are presented.

^2^HF = high forage; HC = high concentrate; S × D = substrate by dilution rate interaction.

^3^Linear response to dilution rate. Lack of superscript 3 indicates non-linear response to dilution rate.

^4^If there were significant (*P* < 0.05) substrate by dilution rate interactions, substrates were compared across each dilution rate: a, b = unlike superscripts indicate significant differences within low dilution rate; p, q = unlike superscripts indicate significant differences within medium dilution rate; x, y = unlike superscripts indicate significant differences within high dilution rate.

^5^ND = not detected.

**Supplementary Table S10.** Relative abundance (% of total 16S rRNA archaeal genes) of archaeal clades in serial rumen cultures growing on a high forage or a high concentrate substrate, at three dilution rates.

| Dilution rate | Low | | Mid | | High | | SEM | Substrate *P* = | Dilution rate *P* = | S × D^1^  *P* = |
| --- | --- | --- | --- | --- | --- | --- | --- | --- | --- | --- |
| Substrate | HF^2^ | HC | HF | HC | HF | HC |  |  |  |  |
| Incubation 1, transfer 8 | | | | | | | | | | |
| Total Methanobacteriales | 2.23 | ND^2^ | 1.41 | 31.0 | 7.02 | 10.6 | 7.23 | 0.12 | 0.19 | 0.12 |
| *Methanobrevibacter* | 2.23 | ND | 1.41 | 31.0 | 7.02 | 10.6 | 7.23 | 0.12 | 0.19 | 0.12 |
| *Methanosphaera* | ND | ND | ND | ND | ND | ND | - | - | - | - |
| Total Methanomicrobiales | 59.4 | 17.9 | 29.9 | 4.76 | 0.75 | 10.6 | 14.4 | 0.15 | 0.14 | 0.26 |
| *Methanomicrobium* | 59.4 | 17.9 | 29.9 | 4.76 | 0.75 | 10.6 | 14.4 | 0.15 | 0.14 | 0.26 |
| Total Methanosarcinales | ND | ND | ND | ND | ND | ND | - | - | - | - |
| Total Methanomassiliicoccales | 38.3 | 82.1 | 68.7 | 64.3 | 92.2 | 78.7 | 16.0 | 0.54 | 0.32 | 0.25 |
| Non-identified archaea | 0.024 | ND | ND | ND | ND | ND | 0.010 | 0.38 | 0.48 | 0.48 |
| Total hydrogenotrophic | 61.7 | 17.9 | 31.3 | 35.7 | 7.77 | 21.3 | 16.0 | 0.54 | 0.32 | 0.25 |
| Total methylotrophic | 38.3 | 82.1 | 68.7 | 64.3 | 92.2 | 78.7 | 16.0 | 0.54 | 0.32 | 0.25 |
| Incubation 2, transfer 4 | | | | | | | | | | |
| Total Methanobacteriales | 22.8 | 5.88 | 49.9 | ND | 5.09 | ND | 8.35 | 0.016 | 0.14 | 0.10 |
| *Methanobrevibacter* | 22.8 | 5.88 | 49.9 | ND | 5.09 | ND | 8.35 | 0.016 | 0.14 | 0.10 |
| *Methanosphaera* | ND | ND | ND | ND | ND | ND | - | - | - | - |
| Total Methanomicrobiales | 7.50 | 15.4 | 3.51 | ND | 0.057 | ND | 4.74 | 0.76 | 0.14 | 0.54 |
| *Methanomicrobium* | 7.50 | 15.4 | 3.51 | ND | 0.057 | ND | 4.74 | 0.76 | 0.14 | 0.54 |
| Total Methanosarcinales | ND | ND | ND | ND | ND | ND | - | - | - | - |
| Total Methanomassiliicoccales | 52.1 | 78.8 | 44.4 | 100 | 69.8 | 100 | 11.0 | 0.007 | 0.42 | 0.44 |
| Non-identified archaea | 17.6 | ND | 2.22 | ND | 25.1 | ND | 9.72 | 0.15 | 0.60 | 0.60 |
| Total hydrogenotrophic | 30.3 | 21.2 | 53.4 | ND | 5.15 | ND | 9.54 | 0.038 | 0.14 | 0.086 |
| Total methylotrophic | 52.1 | 78.8 | 44.4 | 100 | 69.8 | 100 | 11.0 | 0.007 | 0.42 | 0.44 |
| Incubation 2, transfer 8 | | | | | | | | | | |
| Total Methanobacteriales | 5.78 | 32.9 | 20.3 | 67.3 | 50.5 | 100 | 21.9 | 0.075 | 0.16 | 0.88 |
| *Methanobrevibacter* | 5.78 | 32.9 | 20.3 | 67.0 | 50.5 | 100 | 22.0 | 0.076 | 0.16 | 0.88 |
| *Methanosphaera* | ND | ND | ND | 0.32 | ND | ND | 0.25 | 0.45 | 0.50 | 0.50 |
| Total Methanomicrobiales | 33.9 | 41.4 | 17.7 | 32.7 | 6.58 | ND | 20.0 | 0.78 | 0.41 | 0.91 |
| *Methanomicrobium* | 33.9 | 41.4 | 17.7 | 32.7 | 6.58 | ND | 20.0 | 0.78 | 0.41 | 0.91 |
| Total Methanosarcinales | ND | ND | ND | ND | ND | ND | - | - | - | - |
| Total Methanomassiliicoccales | 50.2 | 25.7 | 61.9 | ND | 42.5 | ND | 13.2 | 0.006 | 0.60 | 0.41 |
| Non-identified archaea | 10.2 | ND | ND | ND | 0.46 | ND | 4.40 | 0.41 | 0.49 | 0.49 |
| Total hydrogenotrophic | 39.7 | 74.3 | 38.1 | 99.7 | 57.1 | 100 | 13.5 | 0.005 | 0.43 | 0.61 |
| Total methylotrophic | 50.2 | 25.7 | 61.9 | 0.32 | 42.5 | ND | 13.2 | 0.006 | 0.60 | 0.41 |

^1^S × D = substrate by dilution rate interaction.

^2^ND = not detected.

**Supplementary Table S11.** Comparison of doubling times of methanogens in this and other studies.

| Reference | Methanogen | Origin | Substrates used for methanogenesis | Optimum temperature (°C) | Doubling time (h) |
| --- | --- | --- | --- | --- | --- |
| This study, low dilution rate | Mixed methanogens in rumen mixed cultures with high forage substrate | Rumen, mixed methanogens | H_2_ + CO_2_/ formate/ methyl groups^1^ | 39 | 6.44 -7.09 |
| This study, medium dilution rate |  |  |  |  | 3.38 – 3.53 |
| This study, high dilution rate |  |  |  |  | 1.75 – 1.78 |
| Mathrani and Boone (1985) | Isolate strain SF1 | Salt pond methylotrophic | Methanol/ methylamines | 37 | 10.2 |
| Jarvis et al. (2000) | Isolate BRM9 | Rumen | H_2_ + CO_2_/ formate | 39 | 3.77 |
|  | Isolate BRM16 |  |  |  | 11.2 |
|  | Isolate CM1 |  | H_2_ + CO_2_/ acetate/ methanol/ methylamines |  | 7.81 |
| Hildenbrand et al. (2011) | *Methanosarcina acetivorans* | Marine mud | Methanol/ acetate | 37 | 6 (on methanol) – 49 (on acetate) |
|  | *Methanococcus maripaludis* S2 | Salt marsh and intertidal sediments  H_2_/formate | H_2_ + CO_2_/ formate | 37 | 2 (on formate) |
| Kröninger et al. (2017) | *Methanomassiliicoccus luminyensis* | Human feces | Methanol + H_2_/ methylamines + H_2_ | 37 | 43.2 (on methanol)  50.4 – 55.2 (on methylamines) |
| Khairunisa et al. (2023) | *Methanobrevibacter millerae* | Rumen | H_2_ + CO_2_/ formate | 39 | 5.4 – 14.6 |
|  | *Methanobrevibacter ruminantium* |  |  |  | 16.8 – 29.4 |
|  | *Methanobrevibacter olleyae* |  |  |  | 14.5 – 15.3 |
|  | *Methanobacterium formicicum* |  |  |  | 2.6 |
|  | *Methanosarcina barkerii* |  | H_2_ + CO_2_/ formate/ methanol/ methylamines |  | 5.4 |

^1^Presumed from the composition of the archaeal community.

^2^Not available

(A)

(B)

(C)

(D)

(E)

(F)

**Supplementary Figure S1A - F.** Mass spectrum of acetic acid, unlabeled (M + 0) (A), 2-^13^C-acetic acid (M + 1) (B), propionic acid, unlabeled (M + 0) (C), 1-^13^C-propionic acid (M + 1) (D), butyric acid, unlabeled (M + 0) (E), and 4-^13^C-butyric acid (M+1) (F).

**Supplementary Figure S2.** Total gas production in 8 transfers of serial rumen cultures growing on a high forage [(A) and (B)] or a high concentrate [(C) and (D)] substrate, at three dilution rates. The experiment was conducted twice: Incubation 1 (A) and (C), and 2 (B) and (D). Substrate (S): *P* = 0.002; Dilution rate (D): *P* < 0.001; Transfer (T): *P* = 0.90; (S × D): *P* = 0.004; (S × T): *P* = 0.96; (D × T): *P* = 0.31; (S × D × T): *P* < 0.001; Incubation (I, random): *P* = 0.51; (I × S × T, random): *P* = 0.010; (I × D × T, random): *P* = 0.035; Sequence [Incubation, random]: *P* = 0.012.

**Supplementary Figure S3.** Evolution of reducing potential relative to the Standard Hydrogen Electrode (SHE) in 8 transfers of serial rumen cultures growing on a high forage [(A) and (B)] or a high concentrate [(C) and (D)] substrate, at three dilution rates. The experiment was conducted twice: Incubation 1 (A) and (C), and 2 (B) and (D). Substrate (S): *P* = 0.023; Dilution rate (D): *P* = 0.33; Transfer (G): *P* = 0.18; (S × D): *P* < 0.001; (S × G): *P* = 0.36; (D × G): *P* = 0.98; (S × D × G): *P* = 0.083; Incubation (I, random): *P* = 0.50; (I × S × G, random): *P* = 0.63; (I × D × G, random): *P* = 0.020; Sequence [Incubation, random]: *P* = 0.31.

**Supplementary Figure S4.** Evolution of ammonium concentration in 8 transfers of serial rumen cultures growing on a high forage [(A) and (B)] or a high concentrate [(C) and (D)] substrate, at three dilution rates. The experiment was conducted twice: Incubation 1 (A) and (C), and 2 (B) and (D). Substrate (S): *P* = 0.048; Dilution rate (D): *P* = 0.067; Transfer (T): *P* = 0.002; (S × D): *P* = 0.22; (S × T): *P* = 0.99; (D × T): *P* = 0.44; (S × D × T): *P* = 0.30; Incubation (I, random): *P* = 0.52; (I × S × T, random): *P* = 0.019; (I × D × T, random): *P* = 0.85; Sequence [Incubation, random]: *P* = 0.22.

**Supplementary Figure S5.** Evolution of isobutyrate production in 8 transfers of serial rumen cultures growing on a high forage [(A) and (B)] or a high concentrate [(C) and (D)] substrate, at three dilution rates. The experiment was conducted twice: Incubation 1 (A) and (C), and 2 (B) and (D). Substrate (S): *P* = 0.16; Dilution rate (D): *P* = 0.45; Transfer (T): *P* = 0.63; (S × D): *P* = 0.010; (S × T): *P* = 0.85; (D × T): *P* = 0.45; (S × D × T): *P* = 0.002; Incubation (I, random): *P* = 0.50; (I × S × T, random): *P* = 0.019; (I × D × T, random): *P* = 0.039; Sequence [Incubation, random]: *P* = 0.41.

**Supplementary Figure S6.** Evolution of 2- and 3-methylbutyrate production in 8 transfers of serial rumen cultures growing on a high forage [(A) and (B)] or a high concentrate [(C) and (D)] substrate, at three dilution rates. The experiment was conducted twice: Incubation 1 (A) and (C), and 2 (B) and (D). Substrate (S): *P* = 0.14; Dilution rate (D): *P* = 0.15; Transfer (T): *P* = 0.72; (S × D): *P* = 0.056; (S × T): *P* = 0.72; (D × T) *P* = 0.79; (S × D × T): *P* = 0.086; Incubation (I, random): *P* = 0.51; (I × S × T, random): *P* = 0.021; (I × D × T, random): *P* = 0.10; Sequence [Incubation, random]: *P* = 0.95.

**Supplementary Figure S7.** Evolution of valerate production in 8 transfers of serial rumen cultures growing on a high forage [(A) and (B)] or a high concentrate [(C) and (D)] substrate, at three dilution rates. The experiment was conducted twice: Incubation 1 (A) and (C), and 2 (B) and (D). Substrate (S): *P* = 0.025; Dilution rate (D): *P* = 0.067; Transfer (T): *P* = 0.88; (S × D): *P* = 0.68; (S × T): *P* = 0.84; (D × T): *P* = 0.62; (S × D × T): *P* = 0.002; Incubation (I, random): *P* = 0.49; (I × S × T, random): *P* = 0.013; (I × D × T, random): *P* = 0.21; Sequence [Incubation, random]: *P* = 0.026.

**Supplementary Figure S8.** Evolution of 4-methylvalerate production in 8 transfers of serial rumen cultures growing on a high forage [(A) and (B)] or a high concentrate [(C) and (D)] substrate, at three dilution rates. The experiment was conducted twice: Incubation 1 (A) and (C), and 2 (B) and (D). Substrate (S): *P* = 0.50; Dilution rate (D): *P* = 0.37; Transfer (T): *P* = 0.20; (S × D): *P* = 0.32; (S × T): *P* = 0.96; (D × T): *P* = 0.20; (S × T × D): *P* = 0.19; Incubation (I, random): *P* = 0.081; (I × S × T, random): *P* = 0.008; (I × D × T, random): *P* = 0.94; Sequence [Incubation, random]: *P* = 0.50.

**Supplementary Figure S9.** Evolution of caproate production in 8 transfers of serial rumen cultures growing on a high forage [(A) and (B)] or a high concentrate [(C) and (D)] substrate, at three dilution rates. The experiment was conducted twice: Incubation 1 (A) and (C), and 2 (B) and (D). Substrate (S): *P* < 0.001; Dilution rate (D): *P* = 0.096; Transfer (T): *P* = 0.18; (S × D): *P* = 0.034; (S × T): *P* = 0.12; (D × T): *P* = 0.66; (S × D × T): *P* = 0.018; Incubation (I, random): *P* = 0.80; (I × S × T, random): *P* = 0.13; (I × D × T, random): *P* = 0.25; Sequence [Incubation, random]: *P* = 0.005.

**Supplementary Figure S10.** Evolution of heptanoate production in 8 transfers of serial rumen cultures growing on a high forage [(A) and (B)] or a high concentrate [(C) and (D)] substrate, at three dilution rates. The experiment was conducted twice: Incubation 1 (A) and (C), and 2 (B) and (D). Substrate (S): *P* = 0.021; Dilution rate (D): *P* = 0.25; Transfer (T): *P* = 0.66; (S × D): *P* = 0.001; (S × T): *P* = 0.93; (D × T): *P* = 0.81; (S × D × T): *P* < 0.001; Incubation (I, random): *P* = 0.003; (I × S × T, random): *P* = 0.026; (I × D × T, random): *P* = 0.016; Sequence [Incubation, random]: *P* = 0.11.

**Supplementary Figure S11.** Evolution of succinate concentration in 8 transfers of serial rumen cultures growing on a high forage [(A) and (B)] or a high concentrate [(C) and (D)] substrate, at three dilution rates. The experiment was conducted twice: Incubation 1 (A) and (C), and 2 (B) and (D). Substrate (S): *P* = 0.085; Dilution rate (D): *P* = 0.19; Transfer (T): *P* = 0.004; (S × D): *P* = 0.48; (S × T): *P* = 0.76; (D × T): *P* = 0.74; (S × D × T): *P* = 0.71; Incubation (I, random): *P* = 0.51; (I × S × T, random): *P* = 0.037; (I × D × T, random): *P* = 0.58; Sequence [Incubation, random]: *P* = 0.028.

**Supplementary Figure S12.** Relationship between acetate production and dihydrogen (H_2_) partial pressure in 8 transfers of serial rumen cultures growing on a high forage and or a high concentrate substrate, at three dilution rates. Blue symbols = high forage; Red symbols = high concentrate. Hollow circles = low dilution rate; Solid circles = mid dilution rate; Triangles = high dilution rate.

**Supplementary Figure S13.** Relationship between methane (CH_4_) production and dihydrogen (H_2_) partial pressure in 8 transfers of serial rumen cultures growing on a high forage and or a high concentrate substrate, at three dilution rates. Blue symbols = high forage; Red symbols = high concentrate. Hollow circles = low dilution rate; Solid circles = mid dilution rate; Triangles = high dilution rate.

**Supplementary Figure S14.** Relationship between propionate production and dihydrogen (H_2_) partial pressure in 8 transfers of serial rumen cultures growing on a high forage and or a high concentrate substrate, at three dilution rates. Blue symbols = high forage; Red symbols = high concentrate. Hollow circles = low dilution rate; Solid circles = mid dilution rate; Triangles = high dilution rate.

**Supplementary Figure S15.** Relationship between butyrate production and dihydrogen (H_2_) partial pressure in 8 transfers of serial rumen cultures growing on a high forage and or a high concentrate substrate, at three dilution rates. Blue symbols = high forage; Red symbols = high concentrate. Hollow circles = low dilution rate; Solid circles = mid dilution rate; Triangles = high dilution rate. Shaded area indicates the 95% confidence band.

**Supplementary Figure S16.** Principal component analysis biplot of fermentation variables in 8 transfers of serial rumen cultures growing on a high forage and or a high concentrate substrate, at three dilution rates. Blue symbols = high forage; Red symbols = high concentrate. Hollow circles = low dilution rate; Solid circles = mid dilution rate; Triangles = high dilution rate. Observations from two incubations with eight transfers each.

**Supplementary Figure S17.** Principal component analysis biplots of bacterial phyla in 8 transfers of serial rumen cultures growing on a high forage and or a high concentrate substrate, at three dilution rates. Blue symbols = high forage; Red symbols = high concentrate. Hollow circles = low dilution rate; Solid circles = mid dilution rate; Triangles = high dilution rate. (A) Incubation 1, transfer 8; (B) Incubation 2, transfer 4; (C) Incubation 2, transfer 8.

**Supplementary Figure S18.** Composition of the bacterial community at the phylum level of the inocula of two incubations of serial rumen cultures.


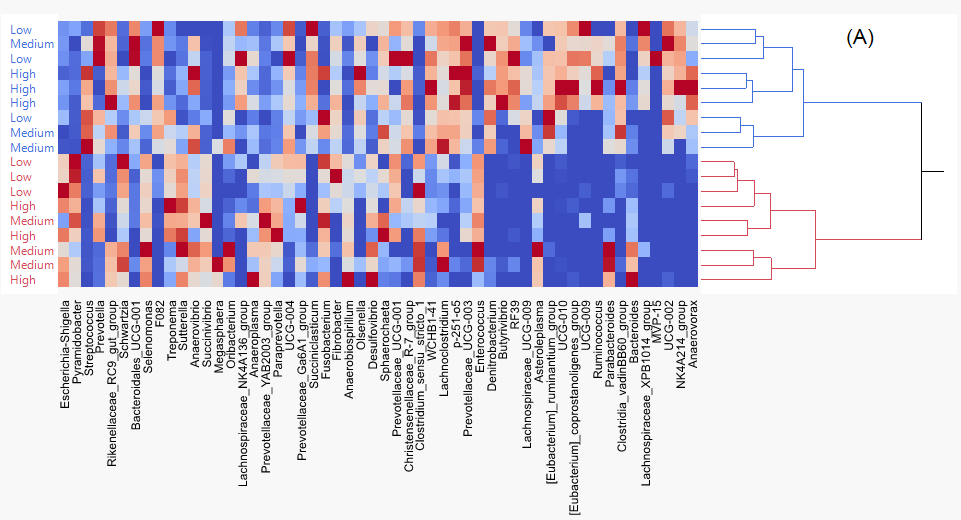


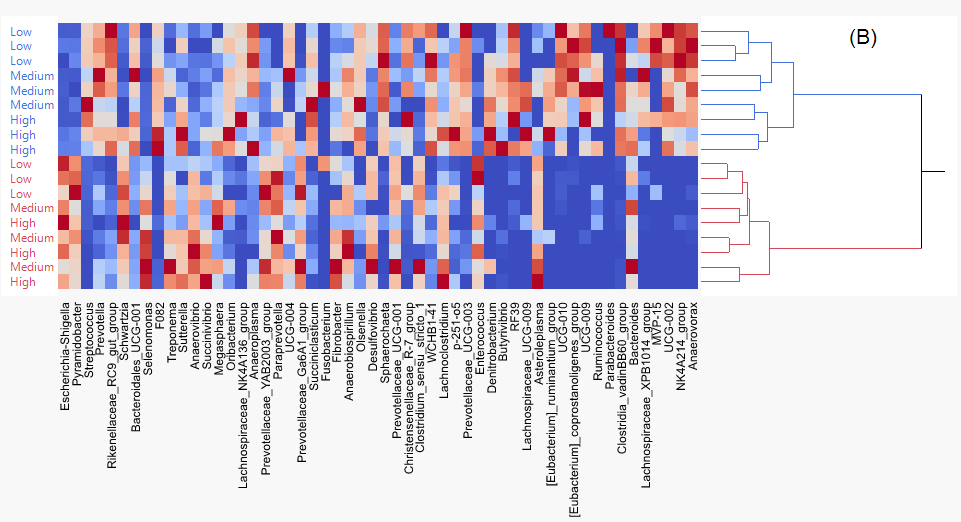


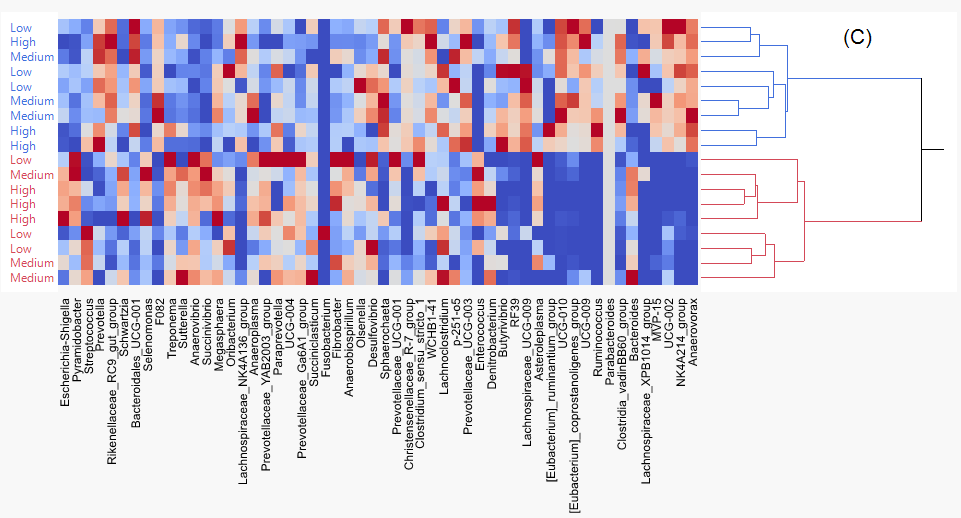


**Supplementary Figure S19.** Cluster analysis of bacterial community composition at genera level of: (A) Incubation 1, transfer 8; (B) Incubation 2, transfer 4, and (C) Incubation 2, transfer 8. In the dendrogram on the right-hand side, high substrate incubations are presented in blue and high concentrate incubations in red. Dilution rates are presented on the left-hand side. Bacterial genera are indicated below each column in the heat map. In the heat map, blue color indicates low relative abundance and red indicates high relative abundance.

**Supplementary Figure S20.** Relative abundance of bacterial genera in the initial inocula.

**Supplementary Figure S21.** Relative abundance of hydrogenotrophic and methylotrophic methanogens in serial rumen cultures growing on a high forage and or a high concentrate substrate at three dilution rates.

**Supplementary Figure S22.** Composition of the archaeal community of the inoculum of two incubations of serial rumen cultures.

**References**

Denman, S.E., Tomkins, N.W., and McSweeney, C.S. (2007). Quantitation and diversity analysis of ruminal methanogenic populations in response to the antimethanogenic compound bromochloromethane. *FEMS Microbiol. Ecol.* 62: 313-322. doi: 10.1111/j.1574-6941.2007.00394.x

Hildenbrand, C., Stock, T., Lange, C., Rother, M., and Soppa, J. (2011). Genome copy numbers and gene conversion in methanogenic archaea. *J. Bacteriol.* 193: 734-743. doi: 10.1128/jb.01016-10

Jarvis, G.N., Strömpl, C., Burgess, D.M., Skillman, L.C., Moore, E.R., and Joblin, K.N. (2000). Isolation and identification of ruminal methanogens from grazing cattle. *Curr. Microbiol.* 40: 327-332. doi: 10.1007/s002849910065

Khairunisa, B.H., Heryakusuma, C., Ike, K., Mukhopadhyay, B., and Susanti, D. (2023). Evolving understanding of rumen methanogen ecophysiology. *Front. Microbiol.* 14: 1296008. doi: 10.3389/fmicb.2023.1296008

Kröninger, L., Gottschling, J., and Deppenmeier, U. (2017). Growth characteristics of *Methanomassiliicoccus luminyensis* and expression of methyltransferase encoding genes. *Archaea* 2017: 2756573. doi: 10.1155/2017/2756573

Maeda, H., Fujimoto, C., Haruki, Y., Maeda, T., Kokeguchi, S., Petelin, M. *et al.* (2003). Quantitative real-time PCR using TaqMan and SYBR Green for *Actinobacillus actinomycetemcomitans*, *Porphyromonas gingivalis*, *Prevotella intermedia*, tetQ gene and total bacteria. *FEMS Immunol. Med. Microbiol.* 39: 81-86. doi: <https://doi.org/10.1016/S0928-8244(03)00224-4>

Mathrani, I.M., and Boone, D.R. (1985). Isolation and characterization of a moderately halophilic methanogen from a solar saltern. *Appl. Environ. Microbiol.* 50: 140-143. doi: doi:10.1128/aem.50.1.140-143.1985

Mesa, V., Gallego, J.L.R., González-Gil, R., Lauga, B., Sánchez, J., Méndez-García, C. *et al.* (2017). Bacterial, archaeal, and eukaryotic diversity across distinct microhabitats in an acid mine drainage. 8. doi: 10.3389/fmicb.2017.01756

Mould, F.L., Morgan, R., Kliem, K.E., and Krystallidou, E. (2005). A review and simplification of the in vitro incubation medium. *Anim. Feed Sci. Tech.* 123-124: 155-172. doi: 10.1016/j.anifeedsci.2005.05.002

Raju, P. (2016). *Homoacetogenesis as an alternative hydrogen sink in the rumen*. PhD thesis, University of Massey. Place published.

Walters, W., Hyde, E.R., Berg-Lyons, D., Ackermann, G., Humphrey, G., Parada, A. *et al.* (2016). Improved bacterial 16S rRNA gene (V4 and V4-5) and fungal internal transcribed spacer marker gene primers for microbial community surveys. *mSystems* 1. doi: 10.1128/mSystems.00009-15
